# Ciliary neurotrophic factor slows axonal transport of signalling endosomes

**DOI:** 10.1093/braincomms/fcag339

**Published:** 2026-09-05

**Authors:** Elena R Rhymes, Cassandra Fetkowitz, Giampietro Schiavo, James N Sleigh, Andrew P Tosolini

**Affiliations:** Department of Neuromuscular Diseases and UCL Queen Square Motor Neuron Disease Centre, UCL Queen Square Institute of Neurology, University College London, London WC1N 3BG, UK; Department of Neuromuscular Diseases and UCL Queen Square Motor Neuron Disease Centre, UCL Queen Square Institute of Neurology, University College London, London WC1N 3BG, UK; Department of Neuromuscular Diseases and UCL Queen Square Motor Neuron Disease Centre, UCL Queen Square Institute of Neurology, University College London, London WC1N 3BG, UK; UK Dementia Research Institute, University College London, London WC1E 6BT, UK; Department of Neuromuscular Diseases and UCL Queen Square Motor Neuron Disease Centre, UCL Queen Square Institute of Neurology, University College London, London WC1N 3BG, UK; UK Dementia Research Institute, University College London, London WC1E 6BT, UK; Department of Neuromuscular Diseases and UCL Queen Square Motor Neuron Disease Centre, UCL Queen Square Institute of Neurology, University College London, London WC1N 3BG, UK; School of Biomedical Sciences, Faculty of Health, Medicine and Behavioural Sciences, The University of Queensland, St Lucia 4072, Australia; Australian Institute for Bioengineering and Nanotechnology, The University of Queensland, St Lucia 4072, Australia

**Keywords:** intravital imaging, motor neurons, neurotrophic factors, neurotrophins, signalling endosomes

## Abstract

Efficient axonal transport is essential for maintaining neuronal function, enabling the bidirectional delivery of diverse cargoes between the cell body and distal compartments. In the neuromuscular system, neurotrophic factors regulate motor neuron survival, function and synaptic connectivity, in part, through retrograde trafficking of activated neurotrophic factor-receptor complexes from the neuromuscular junction to the cell body. We recently demonstrated that brain-derived neurotrophic factor stimulation to muscles selectively enhances retrograde transport of signalling endosomes in fast, but not slow, motor neurons *in vivo*. Moreover, both axonal endosome transport and its brain-derived neurotrophic factor-mediated regulation are disrupted in mouse models of diseases impacting motor neurons, including amyotrophic lateral sclerosis and Charcot-Marie-Tooth disease. Here, we examined whether additional neurotrophic factors, when applied to distal axon terminals, share this transport-modulating property. Through imaging sciatic nerves in anaesthetised mice, we tracked the *in vivo* dynamics of signalling endosomes in fast and slow motor neurons via intramuscular injections of a fluorescent atoxic fragment of tetanus neurotoxin. These injections were co-administered with ciliary neurotrophic factor, hepatocyte growth factor, neurturin, or cleavage-resistant pro-brain-derived neurotrophic factor—four growth factors with known effects on motor neurons. Compared to vehicle-treated controls, pro-brain-derived neurotrophic factor, hepatocyte growth factor and neurturin produced no detectable change in transport dynamics. In contrast, ciliary neurotrophic factor markedly reduced endosome speeds in both fast and slow motor neurons, indicating remarkable selectivity of specific neurotrophic factors in the regulation of signalling endosome transport in motor neurons. Understanding this selectivity may aid the development of muscle-targeted neurotrophic factor-based therapeutic strategies aimed at restoring axonal transport in neurodegenerative disease, peripheral neuropathy and nerve injury.

## Teaser

Rhymes *et al*. report that ciliary neurotrophic factor selectively slows the retrograde transport of signalling endosomes in motor neurons *in vivo*. These findings highlight the specificity with which neurotrophic factors regulate axonal transport and point to a role for this regulation in shaping motor neuron connectivity.

## Graphical Abstract

**Graphical Abstract:**
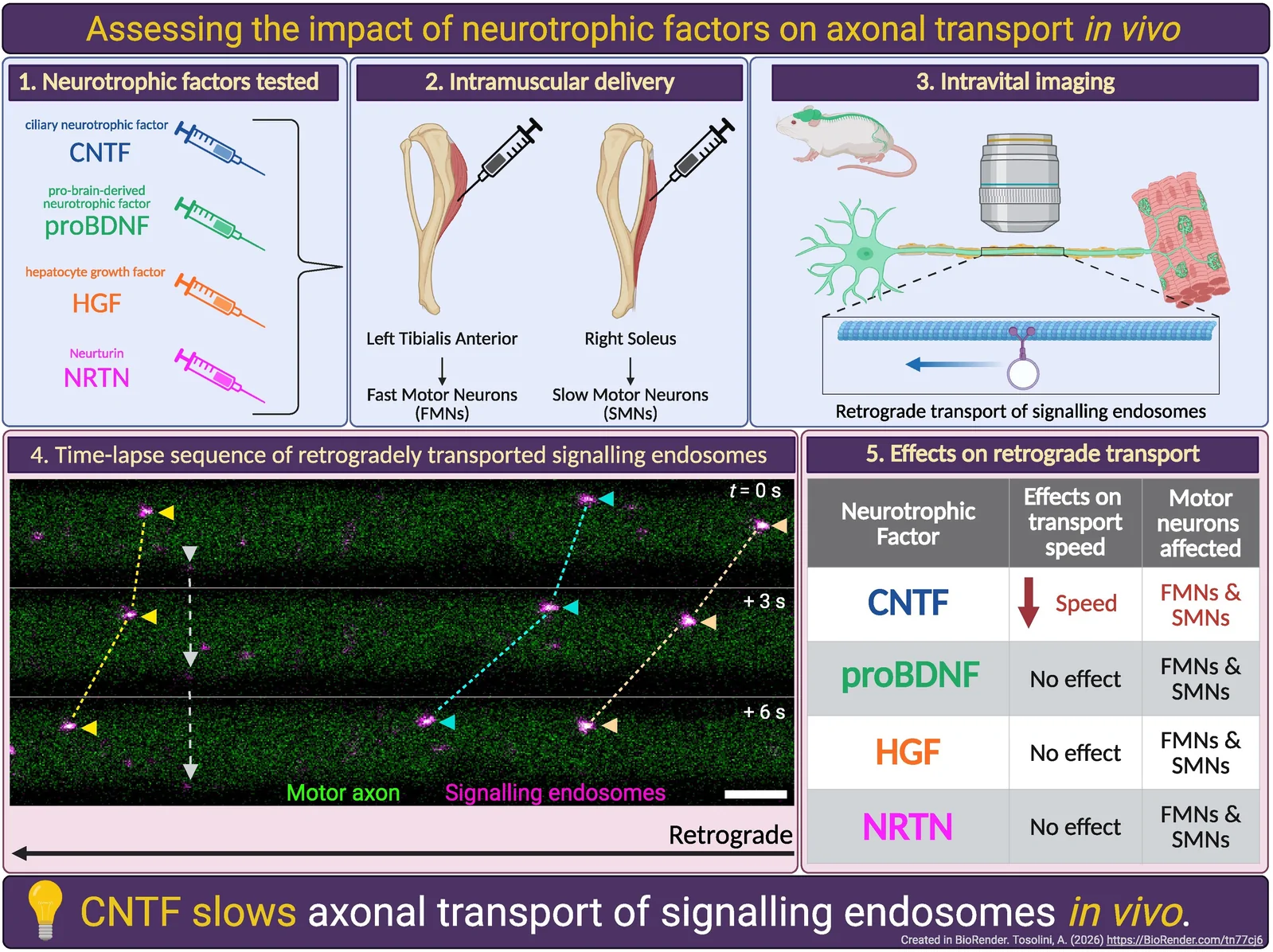

## Introduction

Neurotrophic factors (NTFs) are essential growth factors that regulate the development, maintenance and plasticity of the central and peripheral nervous systems, including the neuromuscular synapse. During embryogenesis, NTFs, including ciliary neurotrophic factor (CNTF), brain-derived neurotrophic factor (BDNF) and neurotrophin-3 (NT-3) guide the survival, differentiation and target innervation of cholinergic motor neurons.^1^ At the neuromuscular junction (NMJ), which is the specialized synapse formed between lower motor neurons and skeletal muscle fibres, bidirectional neurotrophin signalling events support formation, maturation and refinement of the synapse.^2-4^ However, rather than acting uniformly, NTFs exert distinct, context-dependent effects on the NMJ, reflecting a high degree of functional specificity. For example, BDNF and NT-3, but not nerve growth factor (NGF), enhance synaptic activity during development.^5^ Additionally, differences in NTF processing and maturation (e.g. proBDNF versus mature BDNF) can determine receptor and co-receptor binding, further diversifying the influence of NTFs on synaptic and muscle physiology.^6,7^

In adulthood, NTFs and their receptors adapt to the functional demands of the mature nervous system, assuming regulatory roles in synaptic transmission, neuronal excitability, plasticity and regenerative capacity.^8-12^ Teasing apart the embryonic versus mature, context-specific roles of neurotrophic signalling is essential for understanding how these factors shape motor neuron physiology throughout development and into adulthood,^13^ and their potential therapeutic relevance in neuromuscular disorders.

Motor units, comprising α-motor neurons and the skeletal muscle fibres they innervate, are broadly categorized as fast or slow. Fast motor units couple fast motor neurons (FMNs) with fast-twitch type IIb (glycolytic, fatigable), IIx and IIa (oxidative-glycolytic, fatigue-resistant) muscle fibres to produce strong, rapid muscle contractions, whereas slow motor units pair slow motor neurons (SMNs) with slow-twitch type I (oxidative, fatigue-resistant) fibres for sustained, low-force activity.^14,15^ These functional differences are mirrored by distinct motor neuron properties, including size, excitability, metabolic profiles and gene expression.^16-18^

Emerging evidence indicates that NTFs not only support motor neuron survival but also motor unit identity.^19^ Indeed, BDNF within skeletal muscle promotes glycolytic fibre specification,^20^ whereas muscle-secreted neurturin (NRTN) enhances SMN identity.^21^ Such subtype-specific regulation may underlie differential responsiveness to NTF signalling. Notably, increasing the availability of BDNF at motor nerve terminals enhances axonal transport dynamics selectively in FMNs, which exhibit reduced sensitivity to BDNF and impaired cargo trafficking in neurodegenerative conditions [e.g. amyotrophic lateral sclerosis (ALS), Charcot-Marie-Tooth disease (CMT)] and with advanced age.^22-26^

Given the reliance of long-range NTF signalling on intracellular trafficking, axonal transport is central to motor neuron communication and survival.^27^ This microtubule-based process enables neurons to shuttle cargo over long distances, with retrograde transport being crucial for conveying trophic signals from the NMJ to the soma. The signalling endosome serves as the principal vehicle for retrograde transport of NTF-receptor complexes from the NMJ to the motor neuron soma,^28^ and its dynamics can be assessed in both *in vivo* and *in vitro* experimental systems with live fluorescent imaging.^29^ The compartmentalized nature of NTF-receptor interactions suggests that neurotrophins exert spatially distinct effects across motor neuron subtypes. Pioneering this concept, DiStefano *et al*.^30^ demonstrated that BDNF, NT-3 and NGF undergo distinct patterns of retrograde transport in central and peripheral neurons. More recently, Schaller *et al*.^31^ showed that individual NTFs selectively support the survival of discrete embryonic motor neuron subtypes (e.g. CNTF binding to LIFRβ/gp130 for axial motor neurons; hepatocyte growth factor (HGF) binding to mesenchymal–epithelial transition factor (c-MET) for limb motor neurons; and artemin binding to GFRα3-RET for pelvic motor neurons), an effect that exhibited additive impact when applied in combination. These findings emphasize the specificity of NTF signalling and the importance of dissecting their roles in intracellular trafficking and long-range signal propagation in motor neurons.

To assess whether distal neurotrophic cues differentially influence axonal transport across motor neuron subtypes, we selected CNTF, proBDNF, HGF and NRTN for their well-established roles in motor neuron physiology and regeneration. CNTF was selected not only for its function in promoting axonal regeneration^32^ but also due to its widespread use in neuronal cultures to enhance motor axon growth and survival.^23,31,33^ Expanding upon our previous reports that FMN transport is enhanced following BDNF administration,^23^ we selected proBDNF to investigate the effect of specifically engaging p75^NTR^ and sortilin, which are considered to drive largely negative/regressive signalling events.^34,35^ HGF is a pleiotropic growth factor that can be secreted from muscle and promotes embryonic motor neuron survival, with synergistic effects when combined with other NTFs.^31,36^ Finally, NRTN was chosen for its established role in promoting SMN identity and to tease apart MN subtype-specific effects of diverse NTFs.^21^ Accordingly, the receptors for these NTFs have been observed in skeletal muscle and at the NMJ. These include CNTFRα, LIFR and gp130 for CNTF,^37^ RET for NRTN,^38^ the HGF receptor c-MET,^39,40^ and the proBDNF receptors p75^NTR^ and sortilin.^11,23,41^

Using a powerful intravital imaging platform to assess the axonal transport of signalling endosomes in an intact and mature neuromuscular system,^42^ we evaluated whether these four NTFs acutely modulate transport dynamics when delivered to motor nerve terminals. We found that only CNTF influences axonal transport dynamics and did so by reducing mean endosomal speeds.

## Materials and methods

### Ethical approval

All animal procedures were conducted in accordance with the UK Animals (Scientific Procedures) Act (1986) under a license issued by the UK Home Office and were approved by the UCL Queen Square Institute of Neurology Ethical Review Committee.

### Animals

Tg(Chat-EGFP)GH293Gsat/Mmucd mice (RRID: MMRRC_000296-UCD), hereafter referred to as ‘ChAT.eGFP’ mice, were maintained in heterozygosity on a CD-1 background as previously described.^43,44^ Animals were housed in individually ventilated cages under controlled temperature and humidity conditions, with a 12 h light/dark cycle and free access to food and water. Experimental cohorts included both males and females, aged ≈2–3.5 months (mean = 90 days), an interval during which axonal transport dynamics in wild-type mice remain consistent.^24,43^ All procedures and animal care protocols were conducted in accordance with the ARRIVE (Animal Research: Reporting of *In Vivo* Experiments) guidelines to ensure ethical and reproducible research standards.

### *In vivo* axonal transport imaging

**Figure 1:**
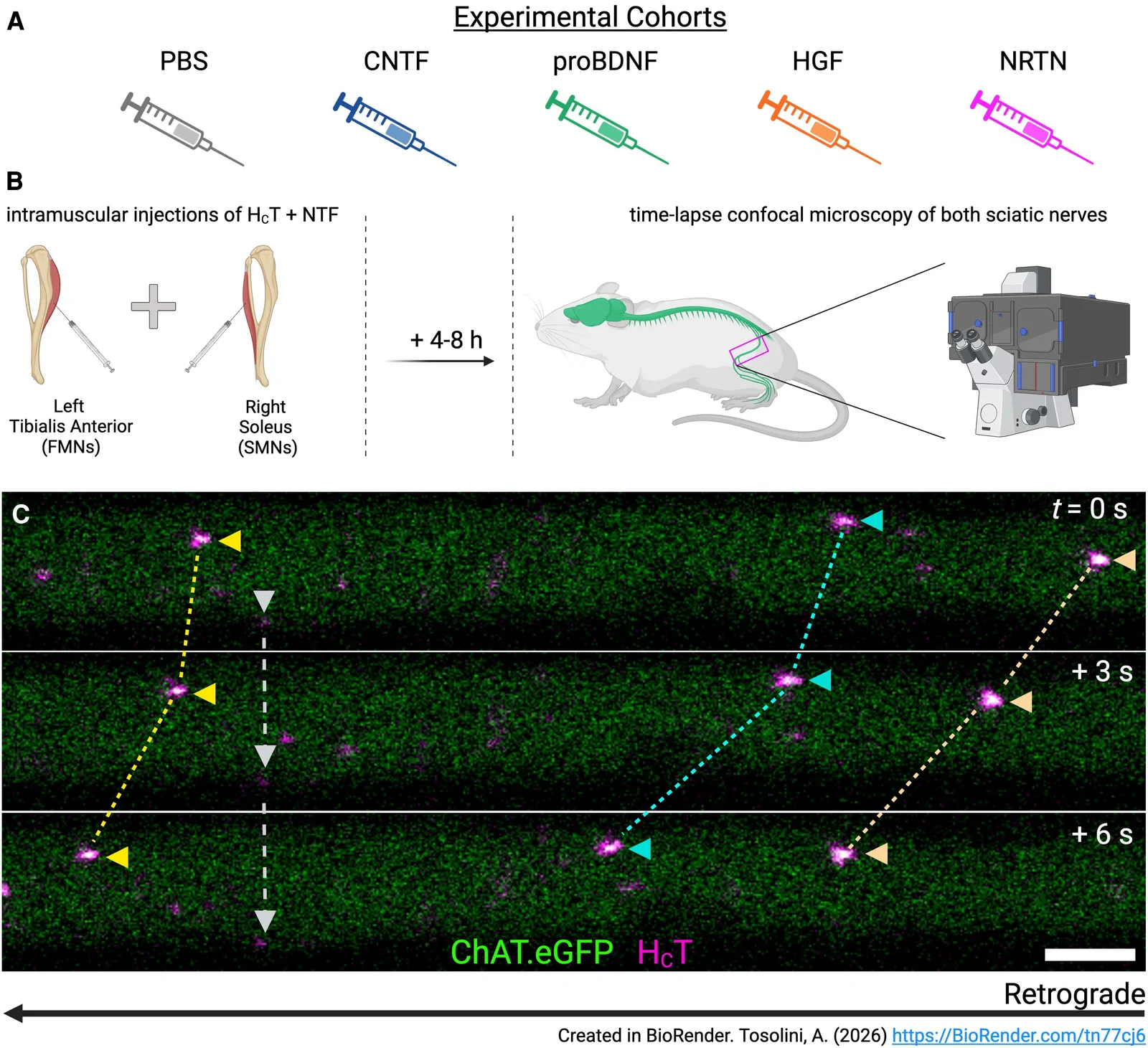
**Experimental design.** (**A)** Male and female choline acetyltransferase enhanced green fluorescent protein (ChAT.eGFP) mice were allocated into one of five groups: (i) phosphate-buffered saline (PBS) control, (ii) ciliary neurotrophic factor (CNTF), (iii) pro-brain-derived neurotrophic factor (proBDNF), (iv) hepatocyte growth factor (HGF), and (v) neurturin (NRTN). (**B)** Each mouse received intramuscular injections of fluorescently conjugated, atoxic binding fragment of tetanus neurotoxin (H_C_T) combined with a single neurotrophic factor (NTF) into the left tibialis anterior and right soleus muscles to target FMNs and slow motor neurons (SMNs), respectively. After a 4–8 h incubation period, time-lapse microscopy was performed on both sciatic nerves. (**C)** H_C_T-labelled signalling endosomes (pseudo-coloured in magenta) from a single ChAT.eGFP motor axon were individually tracked to quantify retrograde transport dynamics. Three representative retrogradely transported signalling endosomes are identified by yellow, cyan and peach arrowheads connected by dashed lines across frames. Grey arrowheads and dashed lines identify a stationary endosome. See also Supplementary Video 1. Scale bar = 5 μm, frame interval = 3 s. Created in parts in BioRender. Tosolini, A. (2026) https://BioRender.com/tn77cj6.

Retrograde axonal transport of signalling endosomes was visualised using an atoxic fragment of tetanus neurotoxin (H_C_T; residues 875–1315), fused to a cysteine-rich domain and a human influenza HA epitope and labelled with AlexaFluor555 C2 maleimide (Thermo Fisher Scientific, A-20346), using reported protocols.^34^ To adhere to the 3Rs principles and to allow direct comparisons between FMNs and SMNs, H_C_T was injected under isoflurane-induced anaesthesia into the left tibialis anterior (TA) and the right soleus (Sol) muscles of the same animal, as previously described (Fig. 1B).^45-48^ Mice were randomly assigned into five experimental cohorts and received 5–7 µg of H_C_T mixed with (i) phosphate buffered saline (PBS) as the control, (ii) 50 ng recombinant human CNTF protein (Peprotech, 450-13), (iii) 50 ng recombinant mouse cleavage-resistant proBDNF (Alomone labs, B-243), (iv) 25 ng recombinant human HGF (Bio-Techne, 294-HG[CF]) or (v) 50 ng recombinant human NRTN (Peprotech, 450-11), as summarized in Fig. 1A. After 4–8 h, mice were re-anaesthetised, and the first sciatic nerve was exposed for imaging. Time-lapse imaging was performed on a Zeiss LSM 780 confocal microscope using a 40× Plan-Apochromat oil immersion objective encased within an environmental chamber maintained at 37°C. All time-lapse images were acquired at intervals within a defined range of 1.0–1.3 s per frame. At the completion of the imaging of the first sciatic nerve, the contralateral sciatic nerve was exposed and imaged. To minimize bias, the order of imaging between the left (TA-injected) and right (soleus-injected) sciatic nerves was randomised. Enhanced green fluorescent protein (eGFP)-positive cholinergic motor axons were imaged, and regions containing nodes of Ranvier were avoided.^43,44^ Following imaging of both sciatic nerves, animals were euthanised within 1 h of terminal anaesthesia induction.

### *In vivo* axonal transport analysis

A minimum of 15 endosomes per axon from at least three axons per mouse were manually tracked using the FIJI plugin ‘TrackMate‘(Fig. 1C and Supplementary Video 1).^49^ Only retrogradely moving signalling endosomes that could be tracked for ≥ 10 consecutive frames were included. An endosome was classified as paused if it remained within an ≈0.2 μm interval for at least two consecutive frames (2 s) to account for minor displacement caused by breathing artefacts. Endosomes were excluded from the analysis if they were immobile for ≥10 consecutive frames (≈10-13 s), or failed to meet the minimum track duration (i.e. they moved for ≤10 consecutive frames). These criteria were chosen to ensure reliable trajectory reconstruction and minimise inclusion of stationary objects that cannot be accurately analysed for transport kinetics. Previous work using this approach has shown that completely immobile endosomes are rare (<1%),^26^ indicating that their exclusion is unlikely to substantially affect the interpretation of transport parameters. The ‘pausing %’ was calculated per animal by determining the number of frames in which endosomes were paused, dividing this by the total number of frames analysed and multiplying this value by 100. Representative kymographs (Supplementary Fig. 2) were generated using the Multi Kymograph plugin (Fiji/ImageJ) from minimally processed time-lapse sequences.

### Protein extraction and western blotting

To confirm activation of downstream signalling, injected TAs were dissected, snap-frozen on dry ice and stored at −70°C. All muscles contained H_C_T + PBS/CNTF/proBDNF/HGF/NRTN, prior to tissue collection. For protein extraction, muscles were thawed on ice, then homogenised in NP-40 lysis buffer [1% (w/v) NP-40, 50 mM NaCl, 50 mM Tris-HCl (pH 8.0)] using Precellys™ tubes containing ceramic beads (VWR, 432-3751). Samples were agitated at 6500 rpm for four intervals of 30 s using the Precellys™ 24 Touch homogenizer in a 4°C cold room. Between intervals, samples were left non-agitating for 90 s to prevent overheating. Homogenates were transferred from the beads to 1.5 ml eppendorfs and centrifuged at 10 000 × *g* for 15 min. A Pierce™ BCA protein Assay Kit (Thermo Fisher) was used to quantify lysate protein concentration. Western blotting was performed as previously described.^23,33^ 60 μg of protein extracts were loaded for each muscle (Supplementary Fig. 5). To determine signalling activation, samples were run twice on parallel blots to quantify total and phosphorylated proteins. Phospho-antibody signals were normalized to total antibody signals, then all values were normalised to the PBS average values to generate percentage activation of PBS. After developing, membranes were incubated with 0.1% Coomassie Brilliant Blue R-250 (Thermo Fisher Scientific, 20278) to assess total protein levels to control for loading. Primary and secondary antibodies used are listed in Supplementary Tables 1 and 2, respectively.

### Immunofluorescence staining of growth factor receptors at the NMJ

To confirm the presence of receptors for CNTF, HGF, proBDNF and NRTN at the NMJ, several different muscle preparations were used. c-MET staining was performed on longitudinal TA sections collected from 1–3 month-old mice transcardially perfused with 4% (w/v) methanol-free formaldehyde (Thermo Fisher Scientific, 28908) in PBS. Muscles were post-fixed for 30 min, washed in PBS, equilibrated in 30% (w/v) sucrose (MilliporeSigma, S7903) in PBS for 24 h, embedded in OCT (361603E, VWR) and cryosectioned at 20 μm. Staining of CNTFRα, GFRα2 and RET was performed in wholemount lumbrical muscles dissected from 1 month-old mice as previously described.^50^ For p75^NTR^ staining, soleus muscles from 3 month-old mice were dissected, post-fixed in 4% formaldehyde for 15–60 min and teased into small fibre bundles prior to staining, as previously described.^23^ All preparations were permeabilised with Triton X-100, blocked in bovine serum albumin-containing buffer and incubated with primary antibodies overnight at 4°C followed by fluorophore-conjugated secondary antibodies. βIII-tubulin (TUJ1) and synaptophysin were used to identify pre-synaptic motor terminals, whereas α-bungarotoxin was used to label post-synaptic acetylcholine receptors. Slides and wholemount preparations were imaged on Zeiss LSM 980 or LSM 780 confocal microscopes using 63× oil immersion and 20× air Plan-Apochromat objectives. NMJ images were acquired as Z-stacks at 1 μm intervals, and secondary-only controls were used to confirm staining specificity. Primary and secondary antibodies are listed in Supplementary Tables 1 and 2, respectively.

### Study design and statistical analysis

Animals were randomly assigned to experimental groups. Treatment allocation was concealed using coded file names during data analysis. All statistical analyses were performed at the conclusion of the tracking. The experimental unit for all transport experiments was the individual animal. Transport measurements were obtained from the left TA and the right Sol muscles within the same animal, and transport assessments from these muscles were analysed separately. Multiple endosomes were tracked per axon, and multiple axons were analysed per animal; however, summary values were calculated per animal for each muscle prior to statistical analysis to account for biological variability between animals. All relevant transport data are summarised in Supplementary Table 3. Group sizes were predetermined based on power calculations, previous experience and the variability observed in comparable transport measurements from our previous studies.^23-26,33,43,44,51^ Data were assessed with two-way analysis of variance (ANOVA) tests, or mixed-effects models fitted by restricted maximum likelihood (REML) where group sizes were unequal, followed by Holm–Šídák’s multiple comparisons tests. Means ± standard error of the mean are plotted for all graphs. GraphPad Prism 10 software (version 10.5.0) was used for statistical analyses and figure production.

## Results

### *In vivo* axonal transport of signalling endosomes is similar between motor neuron subtypes and sexes

Our intravital imaging platform enables real-time visualisation of retrograde axonal transport of neurotrophin-containing signalling endosomes using muscle-injected fluorescent H_C_T^45-47^ (Supplementary Video 1). By injecting selected muscles from contralateral hindlimbs in ChAT.eGFP mice, we compared signalling endosome transport dynamics between FMNs and SMNs within the same animal (Fig. 1). To minimise age-related variability,^26,52^ all experiments were performed in mice aged approximately 3 months.

**Figure 2:**
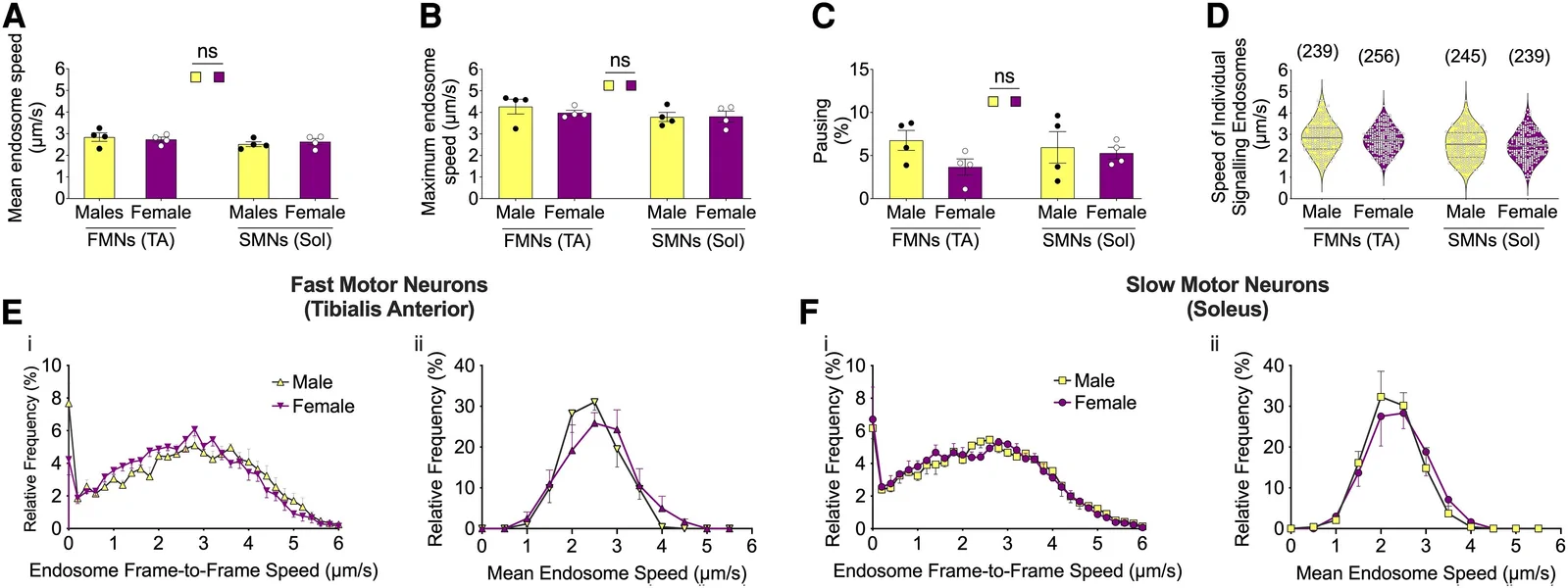
**Under basal conditions, retrograde transport of signalling endosomes is equivalent between male and female mice.** In both fast motor neurons (FMNs) and slow motor neurons (SMNs), males and females exhibit comparable: (**A)** mean endosome speed (two-way repeated-measures ANOVA; motor neuron type: F(1,12) = 2.150, *P* = 0.170; sex: F(1,12) = 0.001, *P* = 0.990; interaction: F(1,12) = 0.580, *P* = 0.460), (**B)** maximum endosome speed (two-way repeated-measures ANOVA; motor neuron type: F(1,12) = 1.760, *P* = 0.210; sex: F(1,12) = 0.290, *P* = 0.600; interaction: F(1,12) = 0.390, *P* = 0.540) and (**C)** pausing endosome percentage (two-way repeated-measures ANOVA; motor neuron type: F(1,12) = 0.106, *P* = 0.750; sex: F(1,12) = 2.340, *P* = 0.150; interaction: F(1,12) = 0.970, *P* = 0.340). Each data point represents one independent animal (*n* = 4 per sex) (**A-C**). (**D)** Violin plots of individual endosomes also indicate similar speed profiles in FMNs and SMNs of both males and females [FMNs: male mean = 2.839 µm/s ± 0.047 (*n* = 239), female mean = 2.728 µm/s ± 0.037 (*n* = 256); SMNs: male mean = 2.522 µm/s ± 0.047 (*n* = 245), female mean = 2.444 µm/s ± 0.042 (*n* = 239)]. The median is shown as a solid grey line, with dashed grey lines indicating the upper and lower quartiles. Furthermore, the endosome frame-to-frame and mean endosome speed distribution curves closely overlap between the sexes for (**E)** FMNs and (**F)** SMNs, with (i) endosome frame-to-frame speed histograms and (ii) mean endosome speed histograms indicating no sex-specific differences in baseline retrograde transport dynamics. Individual animal-level distributions corresponding to these panels are shown in Supplementary Fig. 3A. Statistical analyses for **A–C** were performed using two-way repeated-measures ANOVA with Holm–Šídák's multiple comparisons tests, and no statistical comparisons were performed for **D–F**. The experimental unit for all statistical comparisons was the individual animal (*n* = 4 per sex). ns, not significant.

We first wanted to establish whether baseline transport parameters were similar between sexes and in motor neuron subtypes. H_C_T was therefore injected into the left TA and right Sol, two muscles previously shown to exhibit comparable transport dynamics under basal conditions, yet divergent responses to BDNF stimulation and transport impairments in ALS models.^23,44^ We observed that males and females exhibit similar signalling endosome dynamics in FMNs and SMNs, with no differences in mean speed, maximum speed or pausing events (Fig. 2 and Supplementary Fig. 3A). Therefore, to adhere to the 3R’s principles and minimise animals used in the following experiments, data from males and females were combined.

### CNTF slows retrograde transport in fast and slow motor neurons

**Figure 3:**
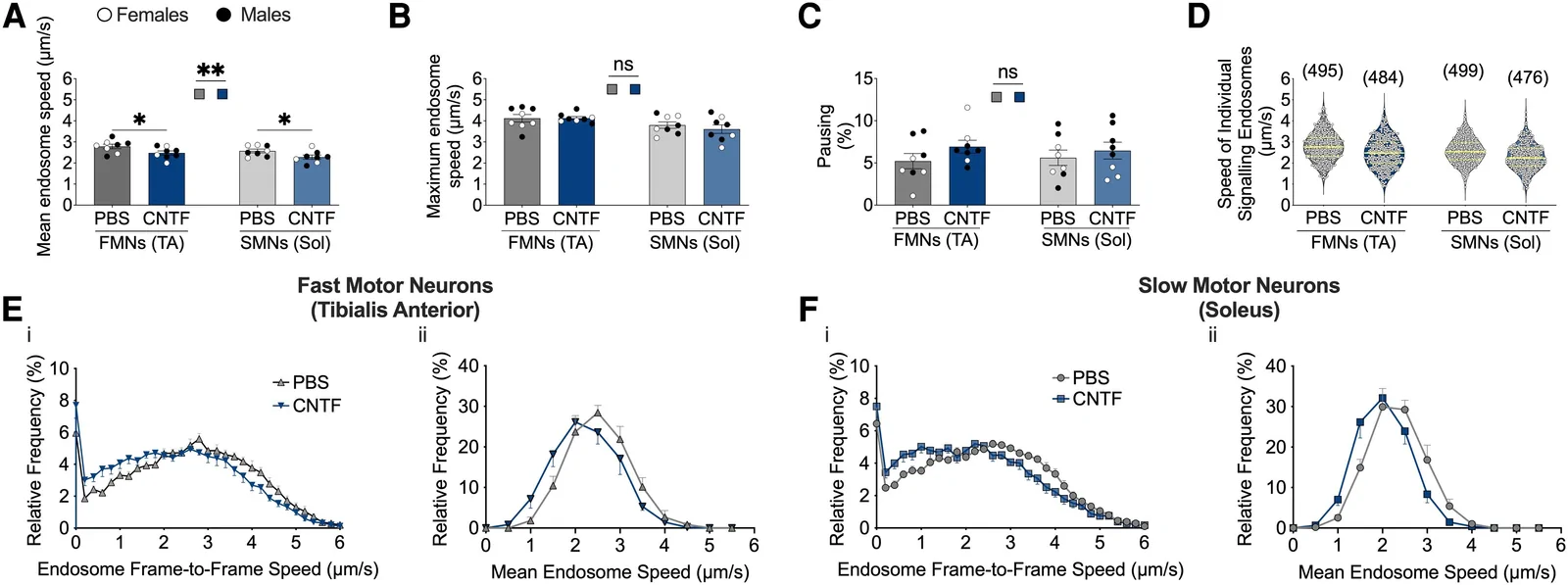
**Acute treatment of muscles with ciliary neurotrophic factor (CNTF) reduces mean signalling endosome transport speed in both fast motor neurons (FMNs) and slow motor neurons (SMNs).** (**A**) Mean endosome speed in both FMNs and SMNs was significantly reduced following CNTF muscle stimulation (two-way repeated-measures ANOVA; stimulation: F(1,14) = 15.700, *P* = 0.001; motor neuron type: F(1,14) = 3.120, *P* = 0.100; interaction: F(1,14) = 0.0098, *P* = 0.920). (**B**) Maximum endosome speed was not significantly affected by stimulation (stimulation: F(1,14) = 0.751, *P* = 0.400; motor neuron type: F(1,14) = 4.620, *P* = 0.050; interaction: F(1,14) = 0.816, *P* = 0.380). (**C**) Endosome pausing percentage was not significantly affected by stimulation (stimulation: F(1,14) = 2.010, *P* = 0.180; motor neuron type: F(1,14) = 0.001, *P* = 0.970; interaction: F(1,14) = 0.230, *P* = 0.640). (**D**) Violin plots of individual endosome speeds show slower transport in CNTF-treated mice [FMNs: phosphate-buffered saline (PBS) mean = 2.782 µm/s ± 0.030 (*n* = 495), CNTF mean = 2.483 µm/s ± 0.032 (*n* = 484); SMNs: PBS mean = 2.570 µm/s ± 0.026 (*n* = 499), CNTF mean = 2.273 µm/s ± 0.028 (*n* = 476)]. The median is shown as a solid yellow line, with dashed yellow lines indicating the upper and lower quartiles. For (**E**) FMNs and (**F**) SMNs, CNTF treatment produced a leftward shift in both (i) endosome frame-to-frame speed histograms and (ii) mean endosome speed histograms, consistent with slower retrograde transport following CNTF exposure. Individual animal-level distributions corresponding to these panels are shown in Supplementary Fig. 3B. Statistical analyses for A**–**C were performed using two-way repeated-measures ANOVA with Holm–Šídák’s multiple comparisons tests, and no statistical comparisons were performed for D-F. The experimental unit for all statistical comparisons was the individual animal (*n* = 8). *< 0.05, **< 0.01. *ns*, not significant. Black circles = males (*n* = 4 PBS; *n* = 5 CNTF), white circles = females (*n* = 4 PBS; *n* = 3 CNTF).

CNTF is a neuropoietic cytokine that promotes motor neuron survival and axonal regeneration via the LIFRβ/gp130 receptor complex and subsequent activation of JAK-STAT3 and ERK1/2 signalling.^32^ It is commonly applied *in vitro* to support axonal growth, can protect vulnerable motor neurons from early degeneration in ALS mice,^53^ can compensate against retrograde deficits observed in the *pmn* model of ALS^54^ and can regulate muscle strength during ageing.^37^ However, the direct effect of CNTF on axonal transport has not been fully elucidated. We first confirmed that CNTFRα (Supplementary Fig. 1A) and associated receptors are present at the NMJ,^55,56^ supporting the potential for local CNTF-mediated signalling within the neuromuscular system. To investigate the effect on transport, we co-injected 50 ng of recombinant CNTF and H_C_T into the left TA and right Sol, followed by intravital imaging of both sciatic nerves 4–8 h later (Fig. 1). Given its overt pro-survival role, we hypothesised that CNTF supplementation would increase transport speeds. Unexpectedly, we found that CNTF did the opposite by reducing mean endosome speeds in both FMNs and SMNs, without influencing maximum speed or pausing (Fig. 3A–D and Supplementary Fig. 2B). This was further supported by a leftward shift in frame-to-frame and mean speed distributions, indicating a global slowing of retrograde transport (Fig. 3E and F and Supplementary Fig. 3B). Importantly, intramuscular delivery of CNTF triggered canonical downstream signalling in the TA, significantly increasing activation of both ERK1/2 and STAT3, but not AKT, confirming receptor engagement *in vivo* (Supplementary Fig. 4A). These observations provide the first evidence that CNTF can negatively modulate, rather than enhance, endosomal transport dynamics, adding a new dimension to its established roles in motor neuron biology.

### proBDNF does not influence retrograde endosomal movement in FMNs or SMNs

**Figure 4:**
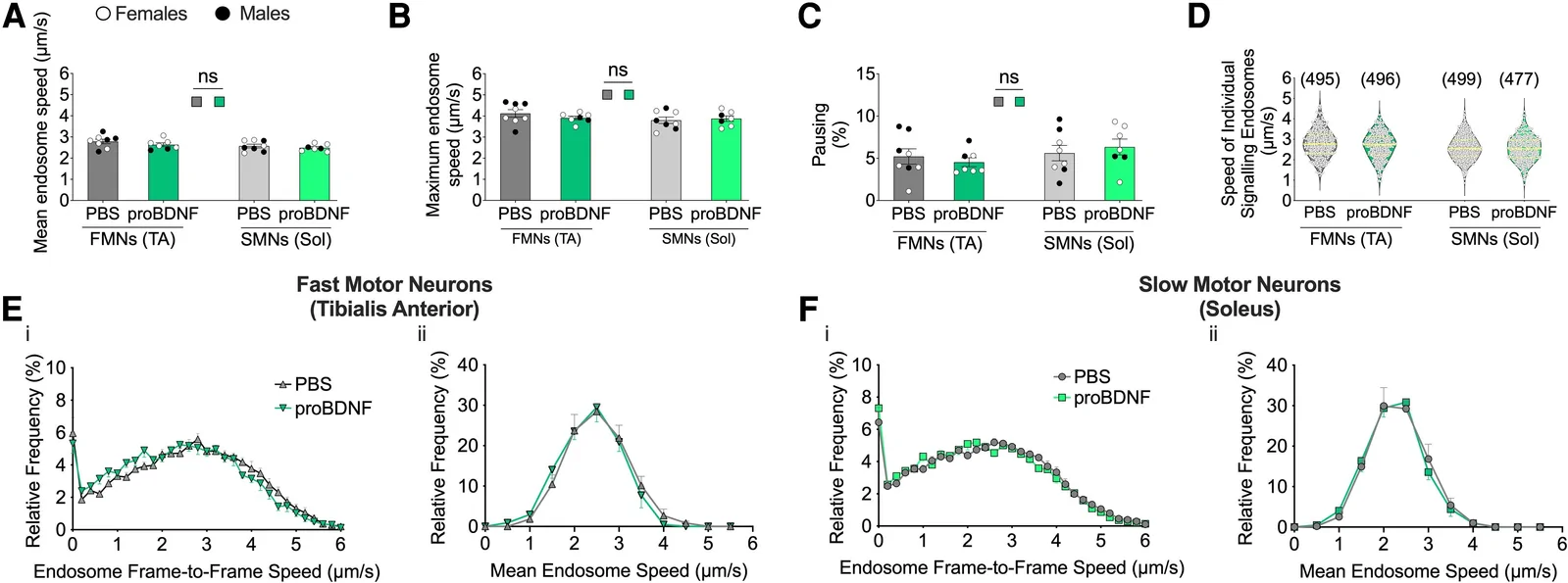
**Stimulation of muscles with pro-brain-derived neurotrophic factor (proBDNF) does not alter the axonal transport of signalling endosomes.** In both fast motor neurons (FMNs) and slow motor neurons (SMNs), proBDNF did not alter: (**A**) mean endosome speed (mixed-effects model, REML; stimulation: F(1,26) = 1.77, *P* = 0.19; motor neuron type: F(1,26) = 4.020, *P* = 0.060; interaction: F(1,26) = 0.148, *P* = 0.700), (**B**) maximum endosome speed (mixed-effects model, REML; stimulation: F(1,12) = 0.254, *P* = 0.620; motor neuron type: F(1,14) = 1.140, *P* = 0.300; interaction: F(1,12) = 1.440, *P* = 0.250) or (**C**) endosome pausing percentage (mixed-effects model, REML; stimulation: F(1,12) = 0.011, *P* = 0.920; motor neuron type: F(1,14) = 1.470, *P* = 0.240; interaction: F(1,12) = 0.986, *P* = 0.340). (**D**) Violin plots of individual endosomes show comparable distributions in all conditions [FMNs: phosphate-buffered saline (PBS) mean = 2.782 µm/s ± 0.030 (*n* = 495), proBDNF mean = 2.670 µm/s ± 0.029 (*n* = 496); SMNs: PBS mean = 2.570 µm/s ± 0.026 (*n* = 499), proBDNF mean = 2.528 µm/s ± 0.027 (*n* = 477)]. The median is shown as a solid yellow line, with dashed yellow lines indicating the upper and lower quartiles. For (**E**) FMNs and (**F**) SMNs, proBDNF treatment resulted in overlapping (i) endosome frame-to-frame speed histograms and (ii) mean endosome speed histograms, consistent with no detectable effect on retrograde transport dynamics. Individual animal-level distributions corresponding to these panels are shown in Supplementary Fig. 3C. Statistical analyses for A–C were performed using mixed-effects models (REML) followed by Holm–Šídák multiple comparisons tests where appropriate, and no statistical comparisons were performed for D–F. The experimental unit for all statistical comparisons was the individual animal (*n* = 7–8). ns, not significant. Black circles = males (*n* = 4 PBS; *n* = 2 proBDNF), white circles = females (*n* = 4 PBS; *n* = 5 proBDNF).

proBDNF is the precursor to mature BDNF and signals primarily through the p75^NTR^-sortilin receptor complex, driving processes such as synaptic pruning and apoptosis.^57^ While mature BDNF elicits pro-survival signalling and selectively enhances axonal transport in motor neurons,^23^ the effects of proBDNF remain unclear. We confirmed localisation of p75^NTR^ to the NMJ (Supplementary Fig. 1B), as has been shown for sortilin, supporting the potential for proBDNF signalling within the neuromuscular system.^11,23,58^ To address this, we injected 50 ng of recombinant, cleavage-resistant proBDNF along with H_C_T into the left TA and right Sol muscles, followed by intravital imaging of the corresponding sciatic nerves 4–8 h later (Fig. 1). We speculated that proBDNF might negatively influence axonal transport through engaging p75^NTR^-sortilin complexes. In contrast, proBDNF stimulation did not alter mean endosome transport speeds, maximum velocities, or pausing events across both FMNs and SMNs (Fig. 4A–D and Supplementary Fig. 2C). Distribution curves of frame-to-frame and mean endosome speeds were largely overlapping with control (Fig. 4E and F and Supplementary Fig. 3C). Despite the absence of transport effects, proBDNF significantly increased activation of ERK1/2, but not AKT, in the TA, confirming biological activity *in vivo* (Supplementary Fig. 4B). Collectively, these data indicate that proBDNF does not modulate retrograde endosomal transport in healthy mice in either motor neuron subtype.

### Axonal transport is not modulated by HGF

**Figure 5:**
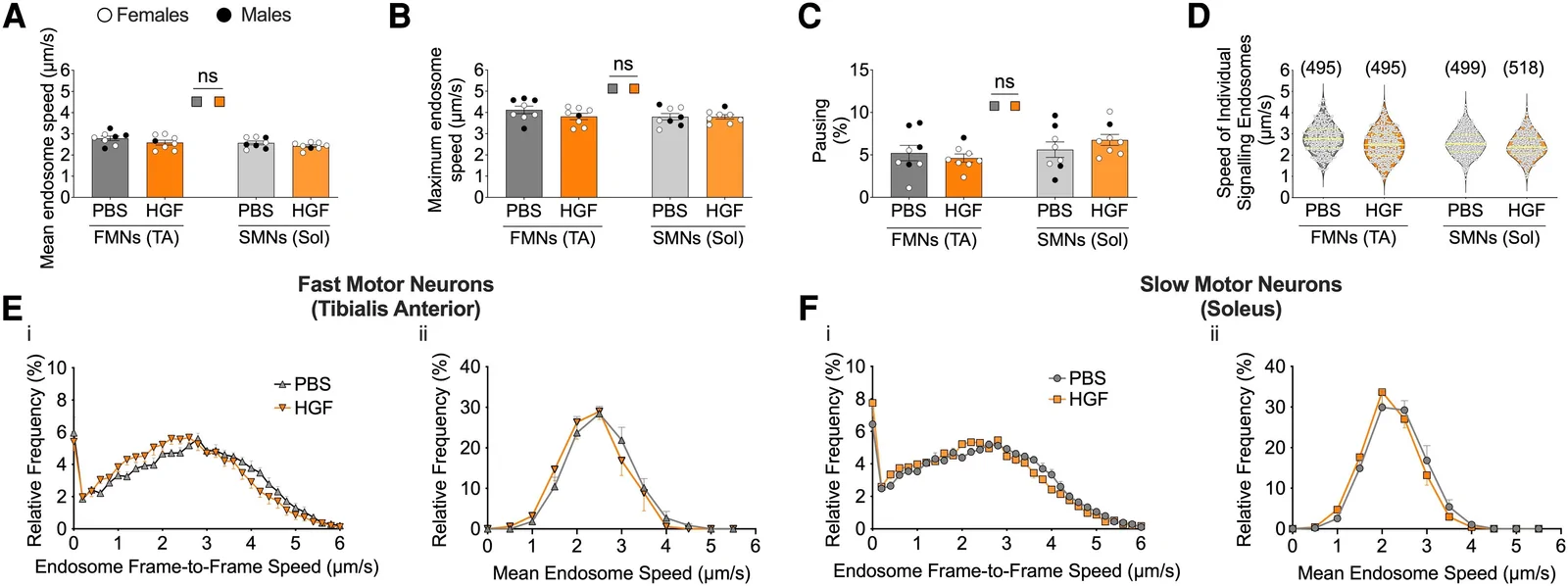
**Stimulation of muscles with hepatocyte growth factor (HGF) does not affect retrograde transport of signalling endosomes.** There were no effects of HGF muscle stimulation across fast (FMNs) and slow (SMNs) motor neurons on (**A)** mean endosome speed (two-way repeated-measures ANOVA; stimulation: F(1,14) = 2.520, *P* = 0.130; motor neuron type: F(1,14) = 6.350, *P* = 0.020; interaction: F(1,14) = 0.048, *P* = 0.830), (**B)** maximum endosome speed (stimulation: F(1,14) = 0.807, *P* = 0.380; motor neuron type: F(1,14) = 2.410, *P* = 0.140; interaction: F(1,14) = 0.766, *P* = 0.400), or (**C)** endosome pausing frequency (stimulation: F(1,14) = 0.133, *P* = 0.720; motor neuron type: F(1,14) = 2.760, *P* = 0.120; interaction: F(1,14) = 1.330, *P* = 0.270). (**D)** Violin plots of individual endosome speeds further demonstrate the similarity between PBS- and HGF-treated motor neurons [FMNs phosphate-buffered saline (PBS) mean = 2.782 µm/s ± 0.030 (*n* = 495), HGF mean = 2.493 µm/s ± 0.030 (*n* = 495); SMNs PBS mean = 2.570 µm/s ± 0.026 (*n* = 499), HGF mean = 2.422 µm/s ± 0.025 (*n* = 518)]. The median is shown as a solid yellow line, with dashed yellow lines indicating the upper and lower quartiles. For (**E**) FMNs and (**F**) SMNs, HGF treatment resulted in substantial overlap between *i*) endosome frame-to-frame speed histograms and *i*i) mean endosome speed histograms, indicating no detectable modulation of retrograde transport dynamics. Individual animal-level distributions corresponding to these panels are shown in Supplementary Fig. 3D. Statistical analyses for **A-C** were performed using two-way repeated-measures ANOVA with Holm–Šídák's multiple comparisons tests, and no statistical comparisons were performed for **D–F**. The experimental unit for all statistical comparisons was the individual animal (*n* = 8). *ns*, not significant. Black circles = males (*n* = 4 PBS; *n* = 1 HGF), white circles = females (*n* = 4 PBS; *n* = 7 HGF).

HGF, which acts through the c-MET receptor, serves as both a muscle-derived survival factor and chemoattractant during development, with pronounced effects on cervical and lumbar motor neurons innervating limb musculature.^36^ Beyond its individual effects, HGF has been shown to amplify the survival and regenerative responses of embryonic motor neurons when combined with other NTFs, including BDNF, CNTF and NRTN.^31^ In the adult CNS, muscle-derived HGF may act as a retrograde survival factor, supporting cholinergic motor neurons after axonal injury.^59^ We corroborated that c-MET is present at the NMJ (Supplementary Fig. 1C), supporting the potential for local HGF signalling within the neuromuscular system.^60^ To test whether HGF modulates retrograde axonal transport, we co-injected 25 ng of recombinant HGF with H_C_T into the left TA and right soleus muscles, followed by intravital imaging on both sciatic nerves 4–8 h later (Fig. 1). Contrary to our expectations, HGF had no effect on endosomal transport in either FMNs or SMNs, with no change in mean speed, peak velocity, or pause frequency (Fig. 5A–D and Supplementary Fig. 2D). In addition, no notable changes were observed in the distribution curves of frame-to-frame or mean endosome speeds compared to controls (Fig. 5E and F and Supplementary Fig. 3D). Although HGF did not alter transport dynamics, intramuscular delivery increased activation of ERK1/2 and AKT in the TA, confirming biological activity *in vivo* (Supplementary Fig. 4C). These results indicate that acute exposure to HGF does not regulate axonal transport kinetics of signalling endosomes in both motor neuron subtypes.

### Axonal transport is unaffected by short-term NRTN exposure

**Figure 6:**
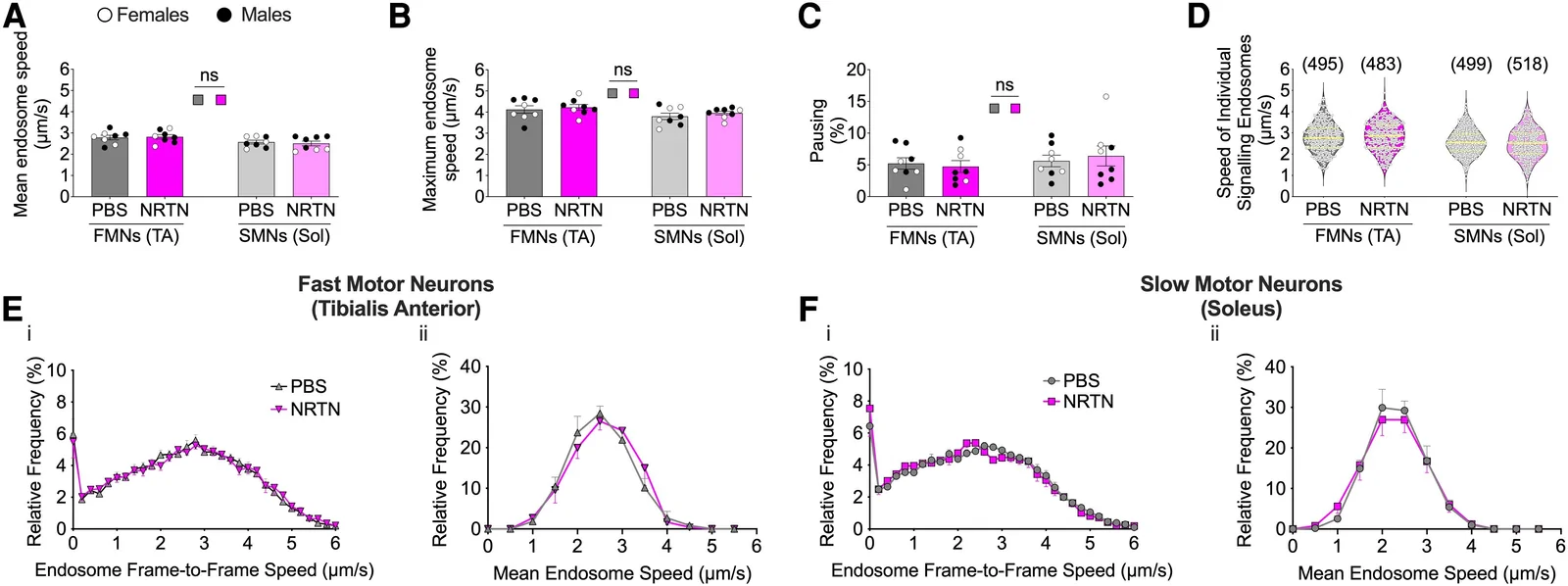
**Stimulation of muscles with neurturin (NRTN) does not alter retrograde transport of signalling endosomes.** Acute NRTN treatment of muscles had no impact on (**A**) mean endosome speed (two-way repeated-measures ANOVA; stimulation: F(1,14) = 0.0250, *P* = 0.880; motor neuron type: F(1,14) = 6.330, *P* = 0.020; interaction: F(1,14) = 0.241, *P* = 0.630), (**B**) maximum endosome speed (stimulation: F(1,14) = 0.880, *P* = 0.360; motor neuron type: F(1,14) = 4.250, *P* = 0.060; interaction: F(1,14) = 0.0370, *P* = 0.850), or (**C**) endosome pausing percentage [stimulation: F(1,14) = 0.039, *P* = 0.850; motor neuron type: F(1,14) = 0.552, *P* = 0.470; interaction: F(1,14) = 0.711, *P* = 0.410). (**D)** Violin plots of individual endosome speeds confirmed that NRTN treatment did not alter transport profiles (FMNs phosphate-buffered saline (PBS) mean = 2.782 µm/s ± 0.030 (*n* = 495), NRTN mean = 2.822 µm/s ± 0.031 (*n* = 483); SMNs mean = 2.570 µm/s ± 0.026 (*n* = 499), NRTN mean = 2.504 µm/s ± 0.031 (*n* = 498)]. The median is shown as a solid yellow line, with dashed yellow lines indicating the upper and lower quartiles. For (**E**) FMNs and (**F**) SMNs, (i) endosome frame-to-frame speed histograms and (ii) mean endosome speed histograms largely overlapped between PBS- and NRTN-treated groups, indicating no effect of NRTN on retrograde transport dynamics. Individual animal-level distributions corresponding to these panels are shown in Supplementary Fig. 3E. Statistical analyses for **A-C** were performed using two-way repeated-measures ANOVA with Holm–Šídák's multiple comparisons tests, and no statistical comparisons were performed for **D-F**. The experimental unit for all statistical comparisons was the individual animal (*n* = 8 animals). *ns*, not significant. Black circles = males (*n* = 4 PBS; *n* = 5 NRTN), white circles = females (*n* = 4 PBS; *n* = 3 NRTN).

NRTN is a member of the GDNF family of ligands that signals through a receptor complex composed of GFRα2 and the tyrosine kinase RET to support the survival and maintenance of neurons.^61^ At the NMJ, NRTN influences both pre- and post-synaptic compartments by promoting motor axon growth, enhancing synaptic vesicle accumulation and stimulating acetylcholine receptor clustering on muscle fibres.^62,63^ More recently, NRTN was identified to be a PGC-1α1-regulated myokine, secreted from skeletal muscle that retrogradely signals to motor neurons to support NMJ formation, motor neuron recruitment and the transcriptional program regulating SMN identity.^21,64^ Importantly, both GFRα2 and RET are present at the NMJ (Supplementary Fig. 1D), with RET also localising to pre-synaptic terminals and modulating axonal transport in ALS models,^33,38^ supporting functional NRTN signalling within the neuromuscular system. To determine whether NRTN directly modulates axonal transport, we co-injected 50 ng of recombinant NRTN with H_C_T into the left TA and right soleus muscles and performed intravital imaging of sciatic nerves 4–8 h later. Despite its role in shaping SMN identity, NRTN had no detectable effects on retrograde transport dynamics; with endosome speeds, velocity distributions and pause frequencies remaining comparable to controls (Fig. 6 and Supplementary Figs 2E and 3E). Although ERK1/2 and AKT activation were not significantly altered by NRTN treatment (Supplementary Fig. 4D(i–iii)), the levels of total phosphorylated ERK1/2 (Supplementary Fig. 4D(iv)) and AKT (Supplementary Fig. 4D(v)) were significantly increased, consistent with downstream pathway activation *in vivo*. These results suggest that NRTN does not acutely modulate axonal trafficking in mature motor neurons *in vivo*.

## Discussion

NTFs are well-established regulators of motor neuron survival, identity and neuromuscular connectivity. Our previous work demonstrated that BDNF selectively enhances signalling endosome transport in FMNs, but not SMNs, in healthy adult mice.^23^ Here, we expanded our analysis to additional NTFs with unknown effects on the axonal transport of signalling endosomes in motor neurons. Surprisingly, none of the four tested NTFs enhanced retrograde transport speeds; proBDNF, HGF and NRTN showed no overt effect, whereas CNTF slowed down endosome transport in both FMNs and SMNs. These findings reveal that the modulatory capacity on axonal transport is not a universal feature of NTFs and may depend, at least in part, on both the ligand and motor neuron subtype.

### Defining the endosomal population

We use the term ‘signalling endosome’ to describe a heterogeneous population of NMJ-derived, H_C_T-labelled and largely Rab7-positive endosomes representing a signalling- and transport-competent subset of retrogradely transported organelles that may or may not contain neurotrophins and/or their receptors.^28,65^ H_C_T, the atoxic binding fragment of tetanus neurotoxin, is endocytosed at the NMJ through an NTF-independent pathway in which extracellular matrix nidogens engage their neuronal binding partners LAR and PTPRδ, directing the neurotoxin complex into axonal signalling endosomes.^66-68^ Accordingly, these endosomes should therefore be interpreted as a common signalling-competent population, rather than receptor-defined endosomal populations associated with individual NTFs. Given this ligand-independent uptake mechanism, incorporation of H_C_T is expected to occur uniformly across experimental conditions. Consistent with this, comparable abundances of H_C_T-positive endosomes were observed across all treatment conditions (e.g. Supplementary Fig. 2), indicating that NTF application did not measurably alter H_C_T entry into the retrograde pool. Therefore, it is unlikely that the differential transport effects observed here arise from uneven distribution of the probe into neurotrophin-specific endosomal populations. Thus, our interpretations relate to the regulation of transport dynamics within a shared signalling-competent endosomal population rather than NTF-specific trafficking events.

### Mechanistic insights from BDNF

Our findings indicate that only CNTF significantly alters signalling endosome transport, highlighting that regulation of trafficking is ligand-specific rather than a universal consequence of receptor activation at the NMJ. Previous studies of BDNF provide a useful mechanistic framework to interpret these ligand-dependent effects, as BDNF has been shown to upregulate endocytosis, enhance endosomal flux, increase retrograde transport speeds and drive transcriptional and translational changes in the soma.^23-25,69-71^ Importantly, the pro-survival actions of NTFs depend not simply on their presence in the soma, but on their successful delivery as activated receptor–ligand complexes from the axon.^72^

This process begins at the distal axon, where BDNF-TrkB engagement activates ERK1/2 and AKT pathways and induces Ca^2+^ influx,^73,74^ while Rab10 contributes to delivery of TrkB into peripheral compartments necessary for the biogenesis of signalling endosomes and propagation of retrograde signalling.^75^ We recently identified that BDNF-dependent activation of ERK1/2 enhances axonal transport by modulating microtubule-associated protein phosphorylation, whereas AKT primarily supports survival signals within the soma.^76^ Indeed, inhibition of axonal AKT does not impair transport, underscoring that not all growth factor-activated pathways promote faster transport.^51^ Consistent with this, BDNF-driven enhancement of trafficking is both transient, dissipating within 24 hours, and ERK1/2-dependent, as sequestration of muscle-derived BDNF or inhibition of ERK1/2 impairs transport.^24^

Trk receptors directly associate with cytoplasmic dynein components, including the 14 kDa light chain (LC) and the 74 kDa intermediate chain (IC).^77^ ERK1/2-mediated phosphorylation of IC facilitates dynein recruitment to signalling endosomes,^78^ while neurotrophin signalling promotes synthesis of cofactors such as Lis1 and p150Glued to fine-tune cargo handling.^79^ In parallel, BDNF-induced Ca^2+^ influx activates calcineurin, dephosphorylating huntingtin and initiating retrograde trafficking.^80^ In BDNF-containing endosomes, glyceraldehyde-3-phosphate dehydrogenase (GAPDH) tethered to the vesicle surface locally generates ATP, supplying the energy required to sustain long-distance transport.^81^ Collectively, this represents a tightly coordinated system integrating receptor signalling, motor activation and local metabolic support. Together, these signalling pathways provide a mechanistic framework to interpret the ligand-specific transport effects observed here, particularly the opposing responses to BDNF and CNTF.

### Divergent NTF effects

Despite converging on common signalling pathways, NTFs vary greatly in their effects on axonal transport. CNTF now joins IGF1^51^ and VEGF-165^24^ as negative regulators of signalling endosome motility. Conversely, proBDNF, HGF and NRTN, similar to NT-3 and NT-4,^24^ exert no detectable influence on motor neuron signalling endosome transport *in vivo.* The case for GDNF is more complex, showing no effect in some mouse models *in vivo*,^23^ whereas *in vitro* assays revealed enhanced motility following GDNF treatment, either alone or with GFRα1, although GFRα1 alone did not elicit an effect.^33^ Whether these NTFs produce additive effects on transport, as observed in embryonic motor neuron survival,^31^ remains unresolved.

An important consideration is that regulation of endosomal axonal transport by NTFs may be dose-dependent, where insufficient local ligand concentration fails to induce signalling, and excessive stimulation can trigger aberrant receptor internalisation and feedback inhibition. Indeed, phosphoproteomic and transcriptomic analyses have shown that prolonged BDNF exposure upregulates several negative regulators of downstream signalling, supporting the concept of dose-dependent feedback control.^76^ Furthermore, administration of 25 ng BDNF has previously been shown to modulate transport *in vivo*,^23-26^ suggesting the existence of an optimal concentration window for effective NTF regulation of trafficking. Accordingly, all NTF doses used here (i.e. 25–50 ng) were intentionally selected to be supraphysiological, with the aim of eliciting robust signalling responses. Although HGF was applied at a lower dose, intramuscular delivery similarly activated canonical ERK1/2 signalling pathways *in vivo*, confirming biological activity and making it unlikely that the lower dose of HGF accounts for the absence of measurable effects on transport.

Although both BDNF and CNTF activate ERK1/2, their opposing transport phenotypes suggest that ERK1/2 activation alone is not sufficient to induce endosomal acceleration. In the case of CNTF, concurrent activation of pathways such as JAK-STAT may differentially regulate ERK-mediated transport responses, resulting in slower endosomal speeds. Pathway bias, therefore, likely dictates effector kinase dominance and shapes motor recruitment, cargo specificity and neuron-type selectivity.^82,83^

A further layer of regulation may stem from the balance between dynein and kinesin motor activities, as previously described.^84^ BDNF signalling may promote a retrograde dominance by increasing dynein recruitment, reducing kinesin drag, or upregulating retrograde-specific transport proteins. Conversely, CNTF may shift the balance the other way around (e.g. by recruiting more kinesin, or reducing the assembly of activated dynein-complexes on signalling endosomes), while non-effectors like proBDNF, HGF and NRTN may leave this equilibrium unchanged. These differences may reflect functional specialisation, with some NTFs optimised for long-distance signalling,^85^ and others for local plasticity, muscle homeostasis^3,20,21,64^ and/or sensory neuron modulation.^86^

### Receptor-level modulation

While the present study focuses on ligands regulating endosomal transport dynamics, unravelling the complexities at the receptor level is equally important. In mature neurons, neurotrophin receptors act as retrograde signalling messengers, transmitting peripheral trophic signals from distal axons to the soma.^87^ Importantly, receptor activation state and endosomal trafficking dynamics, rather than basal receptor abundance alone, are increasingly recognized as key determinants of signalling efficacy. This is particularly relevant for signalling endosomes, which carry neurotrophin receptors, including TrkB and p75^NTR^,^65^ and may therefore integrate ligand-specific signalling responses with distinct transport behaviours. Consistent with this, we established that CNTFRα, p75^NTR^, c-MET, GFRα2 and RET are present at mature NMJs, while ligand-induced activation of downstream signalling events in TA supports functional engagement of these signalling pathways within mature motor units. Transcriptomic data from the *SarcoAtlas* resource^88^ demonstrate that CNTF, proBDNF, NRTN, HGF and their corresponding receptors are expressed in both synaptic and extra-synaptic regions of the adult TA. Interestingly, CNTFRα appears to have an especially important role in motor system development and maintenance, as CNTFRα-null mice exhibit perinatal lethality with severe motor neuron loss, whereas CNTF knockout mice remain viable with comparatively mild phenotypes.^89,90^

Proteomic profiling of H_C_T-labelled endosomes provided further insight into NTF ligand–receptor complexes within retrogradely transported endosomal compartments, detecting CNTFR, LIFR and gp130, also known as IL-6ST (CNTF), Src but not c-MET (HGF), GFRα2 but not RET (NRTN) and p75^NTR^ with Sort1 (proBDNF).^91^ As these datasets were generated *in vitro* from mouse embryonic motor neurons, developmental and physiological differences may influence receptor content relative to mature motor neurons *in vivo*. Nevertheless, these proteomic data provide a useful framework for interpreting ligand-specific effects on retrograde transport. For CNTF, the presence of three major receptor components within H_C_T-labelled endosomes supports canonical receptor complex engagement, while intramuscular CNTF delivery significantly increased ERK1/2 and STAT3, but not AKT activation, suggesting that pathway-specific downstream signalling may contribute to the observed reduction in mean endosome transport speeds. However, the absence of c-MET and RET from embryonic endosomal proteomic datasets,^91^ together with the lack of transport changes following HGF and NRTN stimulation observed in this study, suggests that these pathways may not be directly coupled to regulation of retrograde endosomal transport under basal conditions. However, as RET inhibition enhances transport velocities in an ALS mouse model, but not in healthy mice,^33^ its regulatory influence appears to be context-dependent, resembling previously described disease-associated effects of BDNF signalling.^23-25,92^

Ligand-receptor complex composition represents another determinant of downstream effects of NTFs. For BDNF, varying combinations of the TrkB isoforms (e.g. full length TrkB, truncated TrkB) and p75^NTR^, as homodimeric or heterodimeric complexes, drive distinct signalling outcomes.^57,93-95^ In this regard, we used cleavage-resistant proBDNF to selectively engage p75^NTR^-sortilin signalling pathways; however, acute stimulation with proBDNF did not alter transport dynamics. Albeit initially unexpected, this outcome is consistent with the restricted expression and role of p75^NTR^ in mature uninjured neurons, where its functions are diminished compared to development or injury contexts.^94^ Future studies may benefit from examining p75^NTR^-sortilin modulation in disease settings, where impaired axonal transport could unmask context-dependent effects not evident in healthy neurons.

### Additional considerations

Direct influences of NTFs on axonal transport can also be cargo-specific. For example, IGF1 selectively impairs signalling endosome transport while leaving mitochondrial motility unaffected, underscoring the importance of cargo identity.^51^ Such effects may also extend beyond signalling endosomes via recently described ‘hitch-hiking’ mechanisms. BDNF, for instance, enhances endosome movement yet halts mitochondrial transport through Ca^2+^-dependent interactions with Miro1, favouring local rather than long-range responses.^96^ In addition, BDNF regulates lysosomal trafficking by promoting mTORC1 recruitment to late endosomes,^97,98^ potentially influencing other organelles, such as RNA granules associated with LAMP1-positive vesicles.^99,100^ Rab7-linked alterations reinforce this principle, as they specifically impair signalling endosome trafficking without affecting lysosomal or mitochondrial transport, highlighting that cargo identity critically shapes transport outcomes.^101^ Disentangling these multiple layers of regulation will be key to understand how NTFs differentially regulate cargo trafficking and why BDNF specifically tunes the system toward efficient long-range signalling in motor neurons.

Our study focused on motor neuron axonal transport far from the injection site, but NTF effects may also manifest locally within the injected muscle, at NMJs, or in sensory neurons. Such compartmentalised actions highlight the need for integrated models accounting for spatiotemporal conditions and/or cell specificity. Crucially, signalling outcomes are influenced by receptor context, as the same ligand can act differently when engaging receptors at the soma, axon, or synapse.^102^ Many NTF receptors, including TrkA, TrkC and RET, also act as dependence receptors, triggering alternative signalling when the ligand is absent.^103^ This complexity, together with the varied expression of NTF receptors across muscle and motor neurons, raises important questions about how receptor availability and localisation dictate axonal transport rates and signalling outcomes.

NTF ligand-receptor signalling and transport recruitment are altered in neuromuscular diseases, such as ALS, CMT and following injury.^19,104^ Acute ligand delivery, as used here, may differ markedly from chronic overexpression approaches (e.g. AAV-mediated), which can change receptor availability and feedback control. Indeed, muscle-specific BDNF gene therapy in CMT2D mice restored signalling endosome transport to wild-type levels and increased the motor adaptor Snapin in the sciatic nerve.^24^ Similarly, muscle overexpression of GDNF boosts RET expression and increases ERK/AKT signalling in the spinal cords of ALS mice, suggesting paracrine neuroprotection mediated by retrograde mechanisms.^105^ These findings underscore that transport modulation depends on ligand identity, receptor context and disease state, which are key considerations for therapeutic development.

### Future directions

The cellular mechanisms by which CNTF slows retrograde signalling endosome transport *in vivo* remain incompletely understood. Although CNTFRα was detected at the adult NMJ and intramuscular CNTF delivery activated canonical STAT3 and ERK1/2 signalling pathways *in vivo* without altering AKT phosphorylation, how these pathways selectively yield reduced transport velocities remains unclear. A key priority is therefore to define how receptor engagement at the NMJ alters axonal transport dynamics and whether specific downstream pathways mediate this transport-slowing phenotype. Interestingly, CNTF has been shown to protect vulnerable fast-fatigable motor axons from NMJ denervation and axonal pruning in a time-dependent manner during early disease progression in ALS mice,^53^ raising the possibility that reduced transport dynamics may reflect adaptive or protective signalling responses under specific physiological contexts. Expanding this analysis to additional cargoes, including mitochondria and lysosomes, will clarify whether these effects are cargo-specific and whether anterograde transport is also affected. It will also be essential to assess whether these regulatory pathways remain intact in neuromuscular disorders, such as ALS and CMT, where receptor signalling and axonal transport impairments have been extensively described.

## Conclusions

Our results show that NTF regulation of axonal transport is selective rather than universal. BDNF enhances transport, CNTF slows it down and other NTFs exert no detectable effect, reflecting differences in signalling pathways and receptor interactions. Alterations in axonal transport are therefore highly regulated and not a generic outcome of receptor activation following NTF exposure. Understanding these ligand-, receptor- and context-dependent mechanisms will be critical for designing targeted strategies to fully harness the therapeutic potential of modulating axonal transport in neurodegenerative disease and nerve repair.^106^

## Supplementary Material

fcag339_Supplementary_Data

## Data Availability

The datasets generated and/or analysed for this study are available from the corresponding author on reasonable request.
