# Supplementary material for: Ciliary neurotrophic factor slows axonal transport of signalling endosomes
Source: Brain Commun. 2026 Sep 5;8(5):fcag339. doi: 10.1093/braincomms/fcag339 (PMC13590236; doi:10.1093/braincomms/fcag339)
Supplement: Supplementary file 1 [file fcag339_supplementary_data.zip › Supplementary Video Legend.pdf]

Supplementary Video 1. Representative video of H_CT-containing signalling endosomes (magenta) being retrogradely transported within sciatic nerve motor axons (green). Frame interval: 1.02 s, number of frames: 250, frames per second: 15, acquisition time = 4 min 10 s, scale bar = 5 μm.
