# Supplementary material for: Ciliary neurotrophic factor slows axonal transport of signalling endosomes
Source: Brain Commun. 2026 Sep 5;8(5):fcag339. doi: 10.1093/braincomms/fcag339 (PMC13590236; doi:10.1093/braincomms/fcag339)
Supplement: Supplementary file 1 [file fcag339_supplementary_data.zip › Supplementary_Material.pdf]

Supplementary Material

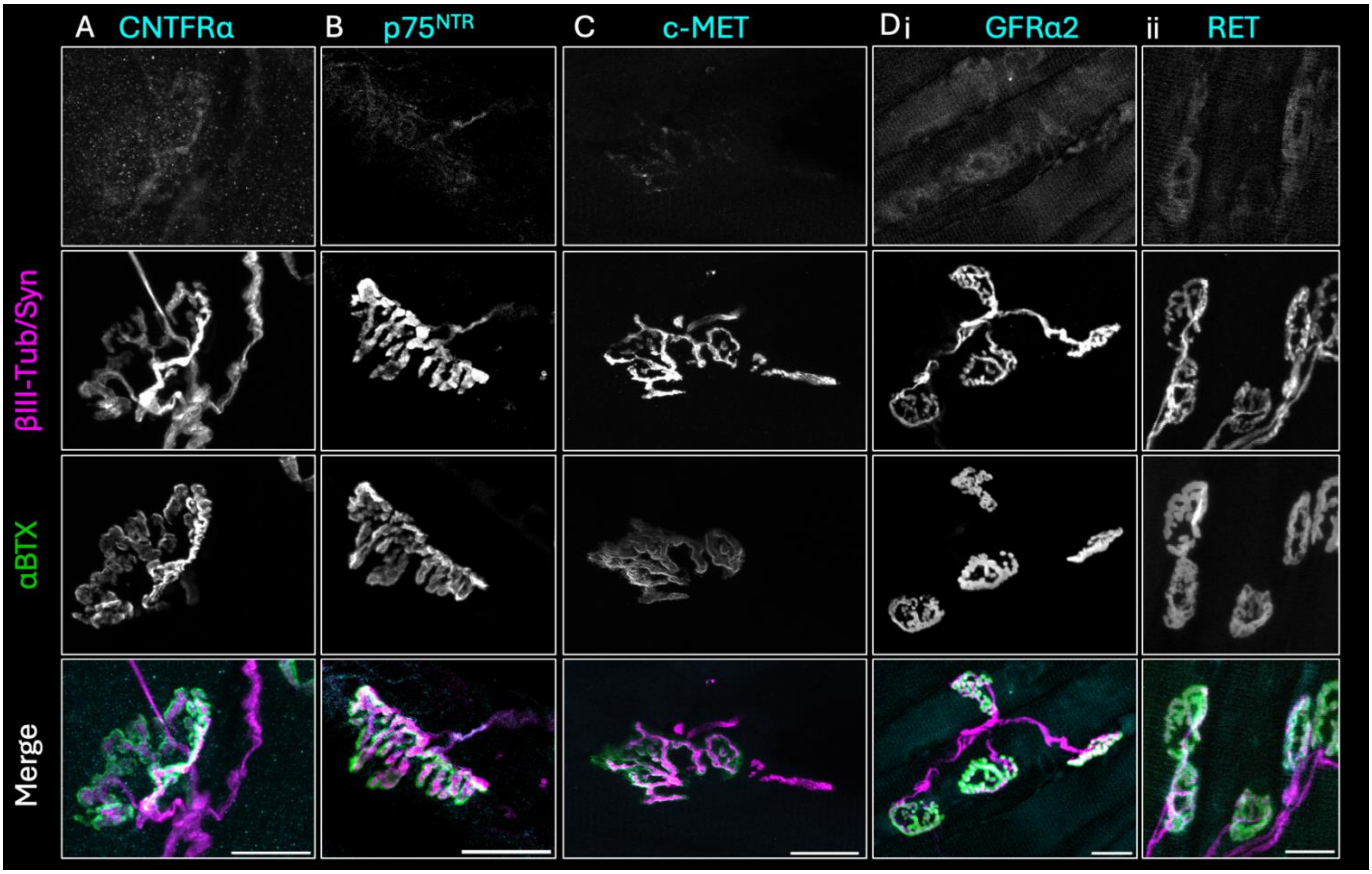

Supplementary Figure 1. Neurotrophic factor receptors at the adult neuromuscular junction. Representative confocal images of adult neuromuscular junctions (NMJs) from either tibialis anterior, soleus or hindlimb lumbricals immunostained for **A**) ciliary neurotrophic factor receptor α (CNTFRα; **B**) p75 neurotrophin receptor ($p75^{NTR}$); **C**) cellular-mesenchymal-epithelial transition factor (c-Met) ; **D***i*) GDNF family receptor α 2 (GFRα2), and **D***ii*) RET. Pre-synaptic motor nerve terminals are labelled with βIII-tubulin/synaptophysin (βIII-Tub/Syn; magenta), and postsynaptic acetylcholine receptors are labelled using α-bungarotoxin (αBTX; green). Top panels show receptor immunoreactivity (white), and bottom panels show merged images illustrating receptor localisation relative to pre- and postsynaptic NMJ compartments. Staining patterns for each receptor were independently confirmed in at least three different mice. Scale bars: 20 μm.

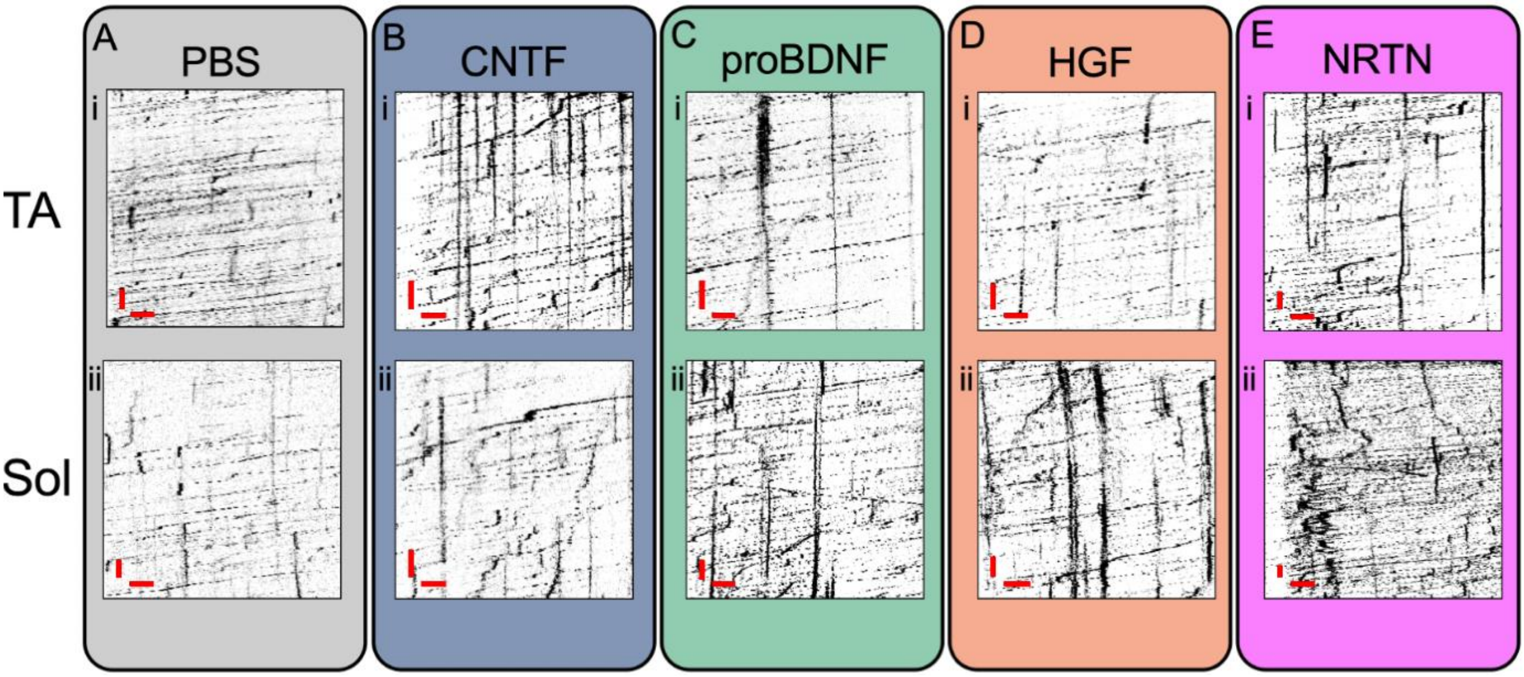

Supplementary Figure 2. Kymographs of *in vivo* axonal transport in motor axons innervating tibialis anterior (TA) and soleus (Sol) muscles from live, anaesthetised mice following neurotrophic factor treatment. Representative kymographs from motor axons innervating the TA (*i*) and Sol (*ii*) following treatment with **A)** phosphate-buffered saline (PBS), **B)** ciliary neurotrophic factor (CNTF), **C)** pro-brain-derived neurotrophic factor (proBDNF), **D)** hepatocyte growth factor (HGF), and **E)** neurturin (NRTN). Retrograde transport is represented by right-to-left downward diagonal tracks, whereas vertical tracks indicate stationary/paused cargoes. Scale bars = 10 μm (horizontal) and 30 s (vertical).

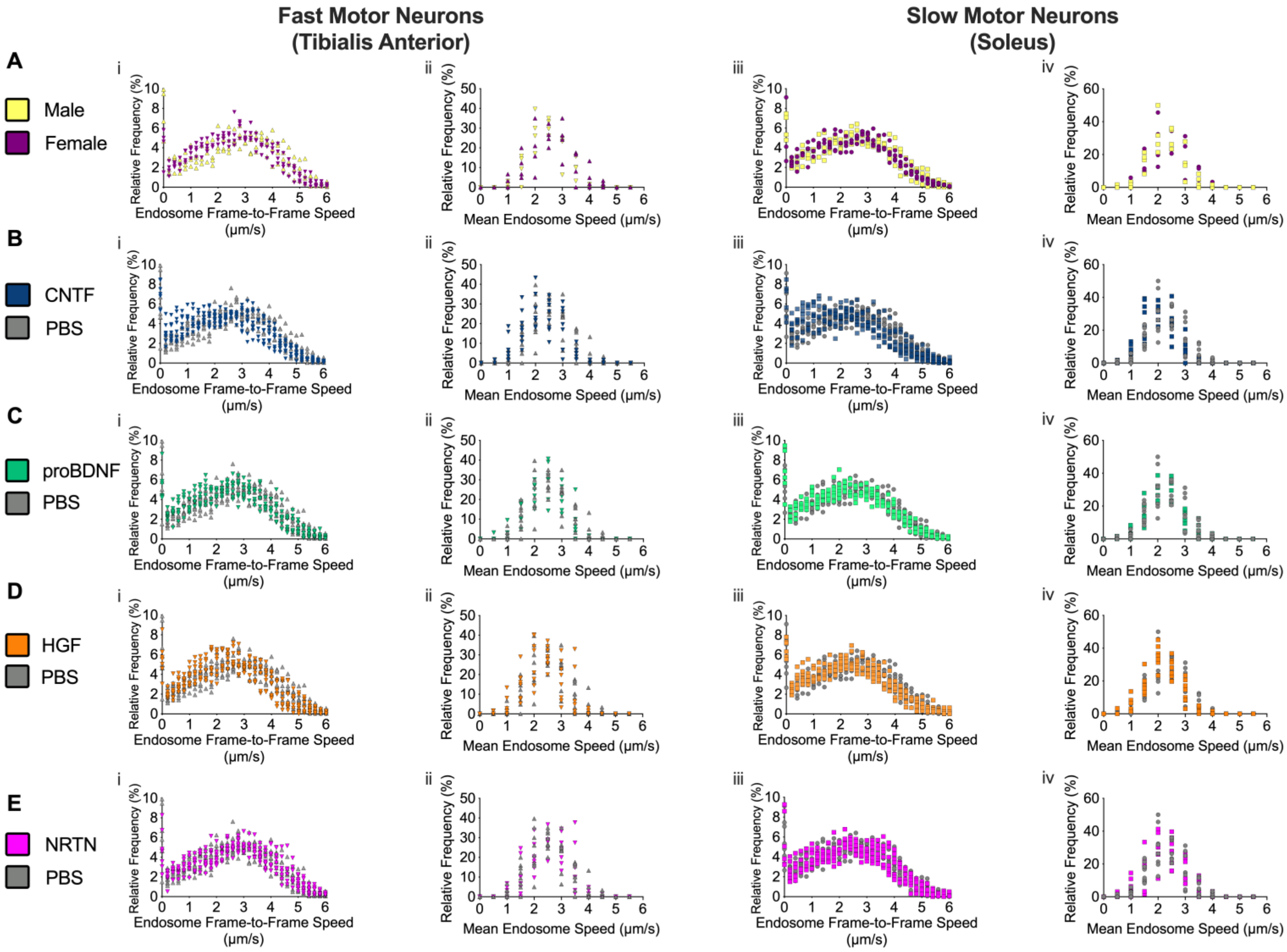

Supplementary Figure 3. Endosome speed distribution plots showing individual animal data corresponding to Figures 2E-F, 3E-F, 4E-F, 5E-F and 6E-F. A) Male versus female in basal conditions. PBS versus **B**) ciliary neurotrophic factor (CNTF); **C**) pro-brain-derived neurotrophic factor (proBDNF); **D**) hepatocyte growth factor (HGF); and **E**) neurturin (NRTN). Within each panel, *i*) frame-to-frame endosome speed distributions for fast motor neurons (FMNs; tibialis anterior, TA), *ii*) mean endosome speed distributions for FMNs, *iii*) frame-to-frame endosome speed distributions for slow motor neurons (SMNs; soleus, Sol), and *iv*) mean endosome speed distributions for SMNs are shown. Each overlaid line represents one independent animal. No statistical comparisons were performed. In panel A, animals are colour-coded by sex (male, yellow; female, purple). In panels B-E, groups are colour-coded as follows: PBS, grey; CNTF, navy; proBDNF, green; HGF, orange; and NRTN, magenta.

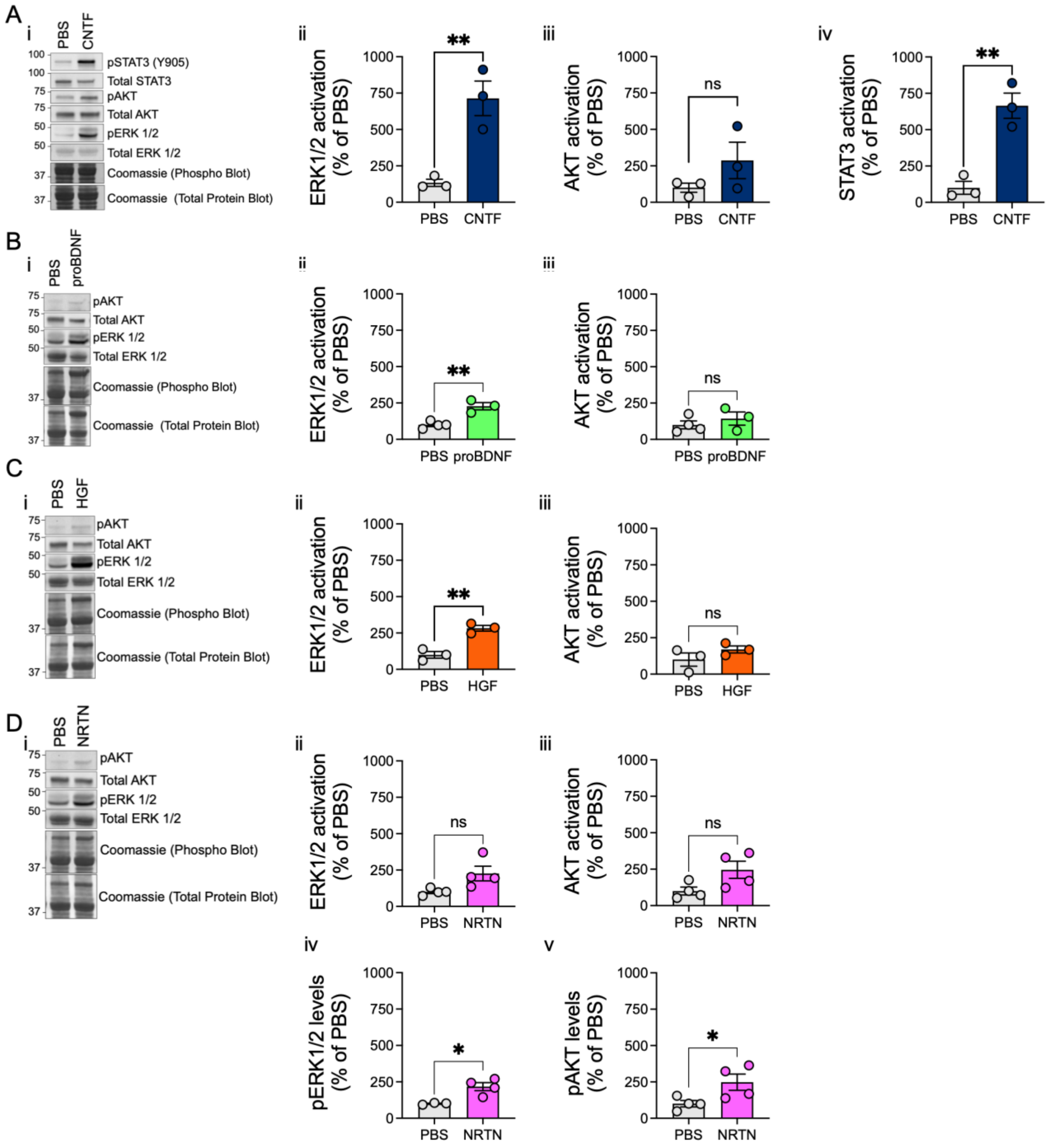

Supplementary Figure 4. Delivery of neurotrophic factors into tibialis anterior (TA) muscle activates canonical signalling pathways *in vivo*. A–D) Representative immunoblots (i) and quantification of downstream signalling pathway activation (ii-v) in TA following intramuscular injection of phosphate-buffered saline (PBS) or the indicated neurotrophic factor. Activation refers to phosphorylated protein levels normalised to corresponding total protein levels, whereas total phosphorylated levels refer to phosphorylated extracellular signal-regulated kinase 1/2 (ERK1/2) or protein kinase B (AKT) normalised only to Coomassie loading controls. **A**) 10-30 min post-ciliary neurotrophic factor (CNTF) treatment there was increased activation of ERK1/2 ($t(4) = 4.803$, $p = 0.009$) and signal transducer and activator of transcription 3 (STAT3) ($t(4) = 5.790$, $p = 0.004$), but not AKT ($t(4) = 1.453$, $p = 0.220$). **B**) 4-5 h post-pro-brain-derived neurotrophic factor (proBDNF) treatment, activation of ERK1/2 ($t(5) = 5.052$, $p = 0.004$), but not AKT ($t(5) = 0.877$, $p = 0.420$), was increased. **C**) 4-5 h post-hepatocyte growth factor (HGF) treatment, ERK1/2 (t(4) = 6.114, $p = 0.004$), but not AKT (t(4) = 1.354, $p = 0.250$), was activated. **D**) 4-5 h post-neurturin (NRTN) treatment there was no significant change in activation of *ii*) ERK1/2 ($t(6) = 2.421$, $p = 0.050$) or *iii*) AKT ($t(6) = 2.258$, $p = 0.060$), although increased total levels of *iv*) phosphorylated ERK1/2 ($t(5) = 3.620$, $p = 0.020$) and *v*) phosphorylated AKT ($t(6) = 2.468$, $p = 0.050$) were observed. For *ii–iv*, each data point represents one independently analysed TA from an individual animal following intramuscular injection of PBS or the indicated neurotrophic factor. All statistical significance was determined using unpaired two-tailed *t*-tests; * $p < 0.05$; ** $p < 0.01$; ns, not significant. n = 3-4.

A CNTF STAT3, AKT and ERK1/2 blots

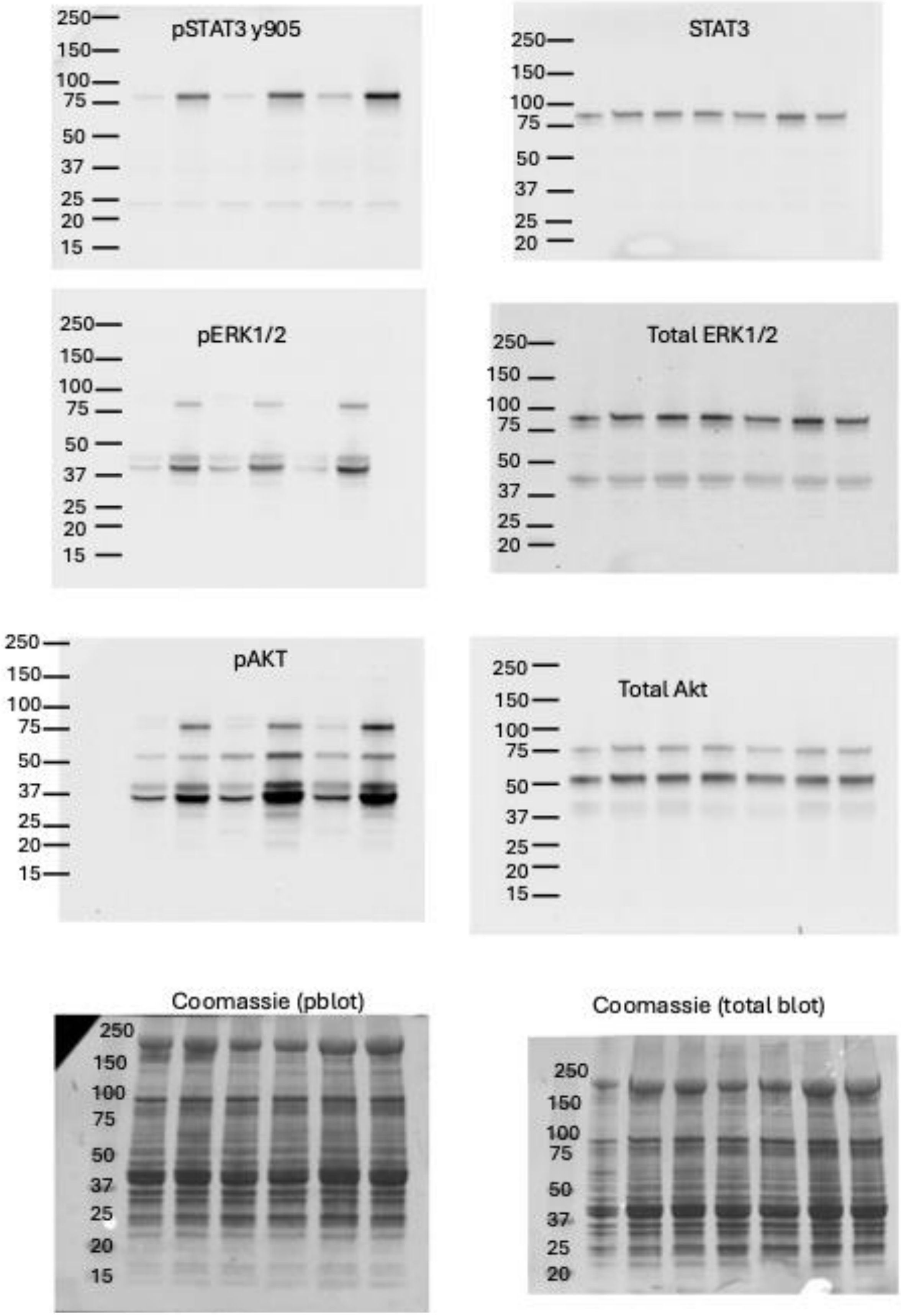

B

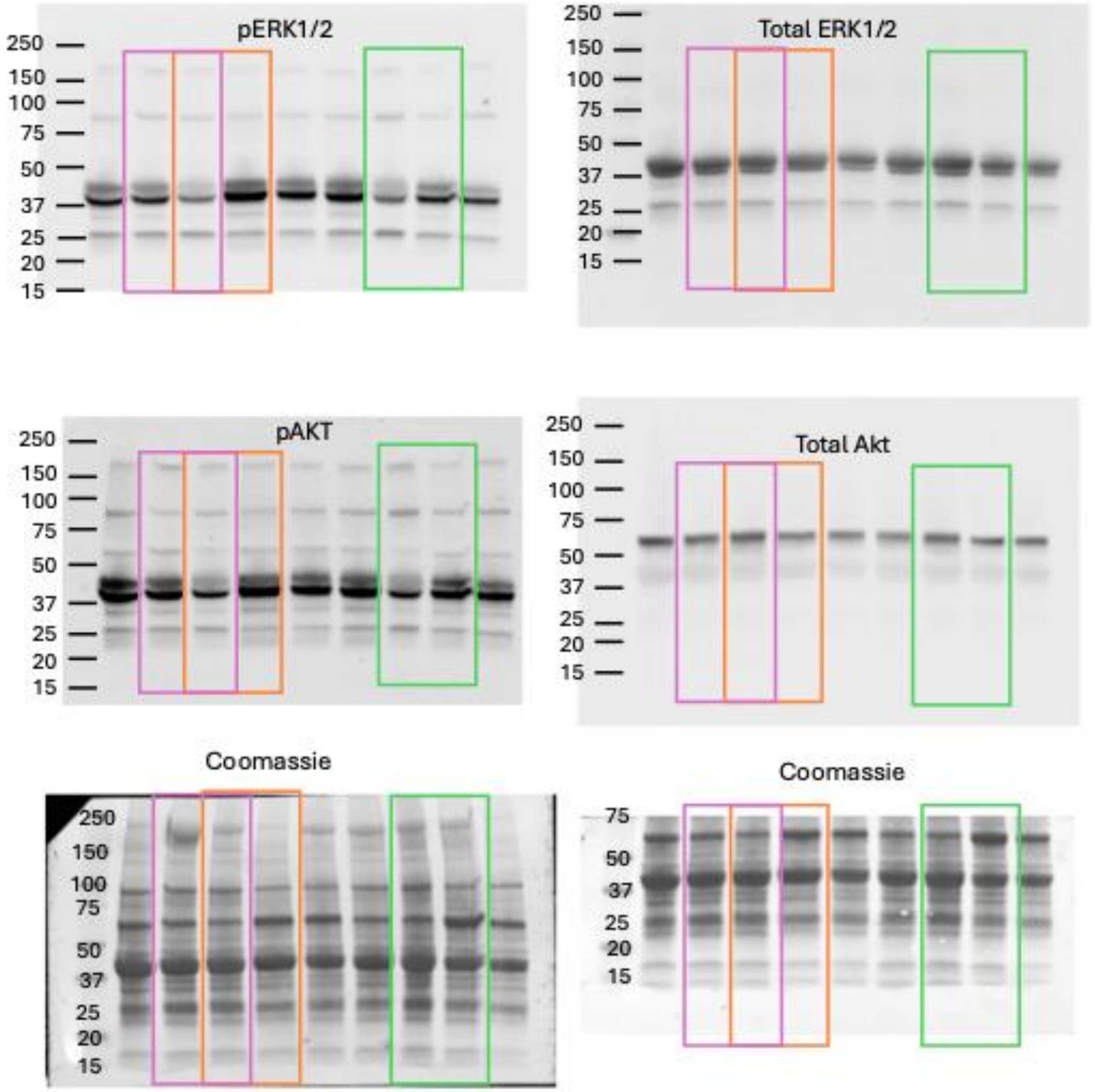

Supplementary Figure 5. Uncropped Western blot images corresponding to Supplementary Figure S4. Full, uncropped Western blot images are shown for **A**) ciliary neurotrophic factor (CNTF) and **B**) pro-brain-derived neurotrophic factor (proBDNF), hepatocyte growth factor (HGF), and neurturin (NRTN) treatment experiments. **A**) Blots from the CNTF experiment show three independent pairs of tibialis anterior muscle samples, with phosphate-buffered saline (PBS)-treated control and CNTF-treated samples presented sequentially. **B**) Blots from the proBDNF, HGF, and NRTN experiments are colour-coded to identify the lanes corresponding to each treatment group, with [green] indicating proBDNF, [orange] indicating HGF, and [magenta] indicating NRTN. Uncoloured regions indicate PBS-control samples. Blots include total and phosphorylated signal transducer and activator of transcription 3 (STAT3), protein kinase B (AKT), and extracellular signal-regulated kinase 1/2 (ERK1/2), together with the corresponding Coomassie-stained loading controls for both the total-protein and phosphoprotein membranes. Where applicable, molecular weight markers are shown in the leftmost lane.

Antibody Target	Dilution	Catalogue Number	Supplier	Method
AKT	1:1,000	9272	Cell Signalling Technology	Western blot
pAKT (S473)	1:1,000	4060	Cell Signalling Technology	Western blot
ERK1/2	1:1,000	9102	Cell Signalling Technology	Western blot
pERK1/2 (T202/Y204)	1:1,000	9101	Cell Signalling Technology	Western blot
STAT3	1:1,000	4904	Cell Signalling Technology	Western blot
pSTAT3 (Y705)	1:1,000	9134	Cell Signalling Technology	Western blot
CNTFRα	1:250	AF-303-NA	R&D Systems	IF: wholemount lumbricals
GFRα2	1:200	AF482	R&D Systems	IF: wholemount lumbricals
HGFR/c-MET	1:250	AF276	R&D Systems	IF: TA sections
$p75^{NTR}$	1:50	G3231	Promega	IF: teased soleus
RET	1:200	AF429	R&D Systems	IF: wholemount lumbricals
Synaptophysin	1:500	MSFR105690	Nittobo Medical	IF: TA sections and wholemount lumbricals
Synaptophysin	1:50	101006	Synaptic Systems	IF: teased soleus
βIII-Tubulin	1:500	MSFR105990	Nittobo Medical	IF: TA sections and wholemount lumbricals
βIII-Tubulin	1:50	302306	Synaptic Systems	IF: teased soleus

Supplementary Table 1. Primary antibodies used in this study. *IF*, immunofluorescence; *TA*, tibialis anterior.

Secondary Antibody	Dilution	Catalogue Number	Supplier	Method
Goat-anti-rabbit IgG (H + L)-HRP conjugate	1:2,000	1706515	BioRad	Western blot
AlexaFluor647 donkey-anti-goat (H+L)	1:500	A21447	Thermo Fisher Scientific	IF: TA sections and wholemount lumbricals
AlexaFluor555 donkey-anti-guinea pig (H+L)	1:500	SAB4600298	Sigma	IF: TA sections and wholemount lumbricals
AlexaFluor488 donkey-anti-goat	1:250	A-11055	Thermo Fisher Scientific	IF: wholemount lumbricals
AlexaFluor488 donkey-anti-guinea pig	1:250	706-605-148	Jackson Immunoresearch	IF: wholemount lumbricals
AlexaFluor488 goat-anti-rabbit (H+L)	1:200	A11034	Thermo Fisher Scientific	IF: teased soleus
AlexaFluor647 goat-anti-chicken (H+L)	1:200	A21449	Thermo Fisher Scientific	IF: teased soleus
AlexaFluor488-conjugated α-bungarotoxin	1:1,000	B13422	Thermo Fisher Scientific	IF: TA sections and wholemount lumbricals
AlexaFluor555-conjugated α-bungarotoxin	1:1,000	B35451	Thermo Fisher Scientific	IF: wholemount lumbricals
AlexaFluor594-conjugated α-bungarotoxin	1:500	B13423	Thermo Fisher Scientific	IF: teased soleus

Supplementary Table 2. Secondary antibodies and fluorescent labels used in this study. *HRP*, horseradish peroxidase; *IF*, immunofluorescence; *TA*, tibialis anterior.

Muscle	Group	Animals	Axons	Signalling Endosomes	Frame-to-Frame Movements	Mean Speed (µm/s)	Max Speed (µm/s)	Pausing (%)
**TA**	PBS	8 (4 ♂/ 4 ♀)	24	495	12,173	2.79	4.12	5.2
**TA**	CNTF	8 (5 ♂/ 3 ♀)	24	484	13,880	2.48	4.13	6.9
**TA**	proBDNF	7 (2 ♂/ 5 ♀)	21	496	12,769	2.63	3.91	4.5
**TA**	HGF	8 (1 ♂/ 7 ♀)	24	495	14,942	2.59	3.81	4.6
**TA**	NRTN	8 (5 ♂/ 3 ♀)	24	483	12,407	2.82	4.23	4.7
**Sol**	PBS	8 (4 ♂/ 4 ♀)	24	499	13,634	2.57	3.80	5.6
**Sol**	CNTF	8 (5 ♂/ 3 ♀)	24	476	13,307	2.28	3.60	6.5
**Sol**	proBDNF	7 (2 ♂/ 5 ♀)	21	477	13,070	2.49	3.87	6.3
**Sol**	HGF	8 (1 ♂/ 7 ♀)	24	518	14,943	2.42	3.79	6.8
**Sol**	NRTN	8 (5 ♂/ 3 ♀)	24	498	13,522	2.51	3.95	6.4

Supplementary Table 3. Sample sizes used in the *in vivo* axonal transport experiments. The number of animals, axons, cargoes and frame-to-frame movements assessed for each *in vivo* axonal transport experimental group are presented. Mean speed, maximum speed and pausing percentage are also provided. *Sol*, soleus; *TA*, tibialis anterior.
